# Isolates from Colonic Spirochetosis in Humans Show High Genomic Divergence and Potential Pathogenic Features but Are Not Detected Using Standard Primers for the Human Microbiota

**DOI:** 10.1128/JB.00272-19

**Published:** 2019-10-04

**Authors:** Kaisa Thorell, Linn Inganäs, Annette Backhans, Lars Agréus, Åke Öst, Marjorie M. Walker, Nicholas J. Talley, Lars Kjellström, Anna Andreasson, Lars Engstrand

**Affiliations:** a Center for Translational Microbiome Research, Department of Microbiology, Cell and Tumor Biology, Karolinska Institutet, Stockholm, Sweden; b Department of Infectious Diseases, Sahlgrenska Academy, University of Gothenburg, Gothenburg, Sweden; c Division for Family Medicine and General Practice, Department for Neurobiology, Care Sciences and Society, Karolinska Institutet, Huddinge, Sweden; d Department of Clinical Sciences, Swedish University of Agricultural Science, Uppsala, Sweden; e Pathology, Aleris Medilab, Taby, Sweden; f University of Newcastle, Callaghan, New South Wales, Australia; g Gastromottagningen City, Stockholm, Sweden; h Stress Research Institute, Stockholm University, Stockholm, Sweden; i Clinical Genomics, Science for Life Laboratory, Stockholm, Sweden; Princeton University

**Keywords:** *Brachyspira*, comparative genomics, spirochetosis, whole-genome sequencing

## Abstract

Colonic spirochetosis, diagnosed based on the striking appearance in histological sections, still has an obscure clinical relevance, and only a few bacterial isolates from this condition have been characterized to date. In a randomized, population-based study in Stockholm, Sweden, 745 healthy individuals underwent colonoscopy with biopsy sampling. Of these individuals, 17 (2.3%) had colonic spirochetosis, which was associated with eosinophilic infiltration and a 3-fold-increased risk for irritable bowel syndrome (IBS). We aimed to culture the bacteria and perform whole-genome sequencing of the isolates from this unique representative population sample. From 14 out of 17 individuals with spirochetosis we successfully isolated, cultured, and performed whole-genome sequencing of in total 17 isolates, including the Brachyspira aalborgi type strain, 513A. Also, 16S analysis of the mucosa-associated microbiota was performed in the cases and nonspirochetosis controls. We found one isolate to be of the species Brachyspira pilosicoli; all remaining isolates were of the species Brachyspira aalborgi. Besides displaying extensive genetic heterogeneity, the isolates harbored several mucin-degrading enzymes and other virulence-associated genes that could confer a pathogenic potential in the human colon. We also showed that 16S amplicon sequencing using standard primers for human microbiota studies failed to detect *Brachyspira* due to primer incompatibility.

**IMPORTANCE** This is the first report of whole-genome analysis of clinical isolates from individuals with colonic spirochetosis. This characterization provides new opportunities in understanding the physiology and potentials of these bacteria that densely colonize the gut in the individuals infected. The observation that standard 16S amplicon primers fail to detect colonic spirochetosis may have major implications for studies searching for associations between members of the microbiota and clinical conditions such as irritable bowel syndrome (IBS) and should be taken into consideration in project design and interpretation of gastrointestinal tract microbiota in population-based and clinical settings.

## Abstract (precis)

This is the first report of whole-genome analysis of clinical isolates from individuals with colonic spirochetosis. This characterization provides new opportunities in understanding the physiology and potentials of these bacteria that densely colonize the gut in the individuals infected. The observation that standard 16S amplicon primers fail to detect colonic spirochetosis may have major implications for studies searching for associations between members of the microbiota and clinical conditions such as irritable bowel syndrome (IBS) and should be taken into consideration in project design and interpretation of gastrointestinal tract microbiota in population-based and clinical settings.

## INTRODUCTION

Colonic spirochetosis (CS) was first described in 1967, when spirochetes adherent to the surface of colonic epithelium were identified by electron microscopy of rectal biopsy specimens in a seminal paper by Harland and Lee, who coined the term intestinal spirochetosis (1). The characteristic histological appearance of the bacteria has been considered to be pathognomonic for diagnosis, but the biology and origin of intestinal spirochetes in humans are still poorly understood.

The nosology for spirochetes in the human intestine has varied from *Borrelia* and *Serpulina* to the present classification *Brachyspira*, and spirochetal bacteria have been identified in the intestines of several animals, including monkeys, dogs, chickens, rodents, and pigs (2). In animals with colonic spirochetosis, a spectrum of medical conditions is well described. While rodents present with asymptomatic excretion, in swine the spirochetes can cause pathological changes leading to diarrhea, malnutrition, and declining growth rates, resulting in high economic losses (3). Primates occupy a rather intermediate position, where even though spirochetal organisms can colonize the colonic mucosa, the animals rarely present with enteric symptoms (4). In humans, at least two spirochete species, Brachyspira pilosicoli and Brachyspira aalborgi, are associated with spirochetosis (5), and rare findings of concomitant infections by B. aalborgi and B. pilosicoli have been described (6, 7). Phylogenetic studies based on the 16S rRNA gene of B. aalborgi made by Pettersson et al. (8) and later confirmed by Mikosza et al. (9), and Westerman et al. (10) have revealed three separate clusters of B. aalborgi: cluster 1, comprising the type strain (513A) and other cultivable strains; cluster 2, suggested as a separate species, “Brachyspira hominis,” by Westerman et al.; and cluster 3, comprising only a few sequences. Also, a cluster 4 was described by Mikosza et al., comprising only sequences from spirochetosis in nonhuman primates.

The spirochetes are both fastidious and rather slow-growing anaerobes (11), and successful culture of spirochetes from stool or biopsy material is rarely reported in human studies. However, the type species of the genus *Brachyspira* (B. aalborgi) was originally isolated from a patient with diarrhea (12), and more recently, another strain isolated from a colonic biopsy specimen in a patient with blood and mucus in stool was reported (13). However, no B. aalborgi and only four B. pilosicoli isolates have been genomically characterized at the whole-genome level to date (14).

Even though there has been progress in detecting and identifying spirochetosis in humans, studies based on histopathological diagnosis without detailed symptom correlation have left the question as to whether it represents a disease process unanswered (15, 16). However, more recent studies have shown an association between spirochetosis and irritable bowel syndrome (IBS) and identified a unique colonic pathology characterized by increased eosinophils under this condition (17, 18). As IBS affects 1 in 10 people worldwide, impairs quality of life, and is highly costly, the observation that colonic spirochetes are associated with IBS diarrhea is of major interest (19).

In a unique representative random population sample that underwent a study using colonoscopy in Sweden, we aimed to culture and perform whole-genome sequencing of colonic spirochetosis isolates obtained from colonic biopsy specimens. To characterize isolates from human spirochetosis, we cultured spirochetal isolates from individuals with microscopically determined spirochetosis from within the randomized, population-based colonoscopy study PopCol, which was performed in Stockholm, Sweden, in 2001 to 2006 (20). A total of 745 healthy adults underwent colonoscopy with biopsy sampling of four sites of the colon and of the terminal ileum. Out of these individuals, 17 presented with spirochetosis, an observation that was shown to be associated with eosinophilic infiltration in the tissue and a 3-fold-increased risk for IBS (17). Since the spirochetes were found solely in the colon (and not in the terminal ileum), we here use the term colonic spirochetosis (3). To further characterize the spirochetal bacteria and their effect on the colonic microbiota, we performed whole-genome sequencing of the bacterial isolates together with the type strain, 513A (ATCC 43994) (12), and reference strain W1 (13), as well as performed 16S amplicon sequencing for microbiota profiling of colonic biopsy specimens in the individuals with colonic spirochetosis and unaffected controls. We present here the first whole-genome sequences of isolates from human colonic spirochetosis, which display extensive genetic heterogeneity between members of the same species.

## RESULTS

### Isolation of spirochetes.

In the present study, spirochetes were successfully isolated from frozen biopsy specimens from 14 out of the 17 individuals (Table 1). All three individuals from whom we were not able to retrieve viable bacteria were in the IBS group. From one of the individuals, colonies of different morphologies were identified and two isolates were propagated, PC5587-p and PC5587-u. In addition to the clinical isolates, the B. aalborgi type strain, 513A (ATCC 43994/NCTC 11492) (12), and the Swedish B. aalborgi reference isolate W1 (13) were sequenced.

**TABLE 1:** Subject and isolate information^*a*^

Patient no. or strain type	Isolate(s)	Sex	Age (yrs)	IBS
1	NC	M	37	IBS-D
2	PC2022III	M	58	
3	PC2777IV	M	45	
4	PC3053II	F	46	
5	PC3517II	F	58	
6	PC3714II	M	60	
7	PC390II	M	34	
8	PC3939II	M	66	
9	PC3997IV	F	31	IBS-M
10	NC	F	27	IBS-U
11	PC4226IV	M	67	
12	PC4580III	F	69	
13	PC4597II	F	36	
14	NC	M	49	IBS-D
15	PC5099IV	F	55	IBS-U
16	PC5538III	M	44	IBS-M
17	PC5587-p, PC5587-u	F	57	
Type strain	513A			
Reference strain	W1			

a NC, not culturable; M, male; F, female; IBS-D, IBS diarrhea; IBS-M, IBS, mixed; IBS-U, IBS, undefined.

### Morphological and biochemical characterization of the isolates.

**FIG 1:**
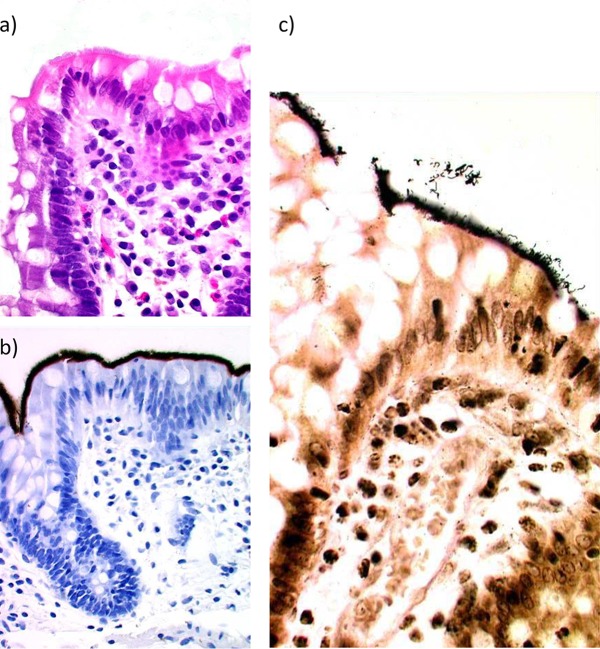
Histological sections showing, in the same formalin-fixed, paraffin-embedded biopsy specimen, representative pictures of spirochetosis using hematoxylin and eosin staining (a), immunohistochemistry using polyclonal spirochete-specific antibodies (b), and Warthin-Starry silver staining (c).

Originally identified in hematoxylin-and-eosin-stained sections and confirmed with immunohistochemistry (IHC), as was previously described by Walker et al. (17), the spirochetes could be observed to completely cover the colonic epithelium in the biopsy specimens of the CS cases. The spirochetosis was verified for this report with new IHC as well as with Warthin-Starry silver stain; representative pictures of the histological manifestation are shown in Fig. 1. The spirochetes were present at all the colon levels studied except for in rectal biopsy specimens, where we could not detect the spirochetes for 4 out of 17 individuals. In the tissue sections the bacteria were of similar morphology in all individuals, with medium-long spiral bacteria seemingly adherent to the colon mucosa. In culture, the growth of the 14 successfully propagated isolates on the fastidious anaerobic agar plates was mostly weak, with small colonies and occasional swarming. The isolates, in contrast to what was observed in the tissue sections, showed a considerable variation in phenotype: growth rate, cell size, and biochemical reactions (Table 2).

**TABLE 2:** Biochemical analyses

Type of isolate or strain	Isolate	Result^*c*^ for:				
		Hemolysis	Indole production	Hippurate hydrolysis	α-Gal^*a*^	β-Glu^*b*^
Clinical isolate	PC4226IV	Weak	−	−	−	+
	PC3053II	None	+	−	−	−
	PC4580III	None	−	−	−	−
	PC3997IV	Weak	−	−	−	−
	PC2777IV	Weak	−	−	−	−
	PC3939II	Weak	+	−	−	+
	PC3517II	Weak	+	−	−	+
	PC3714II	None	−	−	−	+
	PC5099IV	None	−	−	−	−
	PC5538III	None	+	−	−	−
	PC4597II	None	−	−	−	(+)
	PC390II	None	−	−	−	(+)
	PC2022II	None	−	(+)	−	−
	PC5587II-p	None	−	−	−	−
Type strain	513A	None	−	−	−	−
Reference strain	W1	None	−	(+)	−	−

a α-Gal, alpha-galactosidase activity.

b β-Glu, beta-glucosidase activity.

c −, negative; (+), weak positive; +, positive reaction.

Out of the 14 spirochetal isolates that were investigated, 1 isolate shared its reaction pattern with the B. aalborgi reference strain W1 and 5 isolates shared their reaction patterns with the B. aalborgi type strain, 513A (Table 2). Eight strains showed different reaction patterns, including four indole-positive isolates. Two isolates, including the reference strain W1, had a positive but weak hippurate hydrolysis, and five of the strains showed weak hemolytic activity.

### Genome assembly and phylogenetic classification.

Whole-genome *de novo* assembly of the 17 isolates revealed that the bacteria had genomes of approximately the same size, on average 2.67 Mbp, with the smallest being 513A^T^, with a genome of 2.50 Mbp (Table 3). The PC5538III culture turned out to contain two isolates that could be separated in the assembly based on coverage over the contigs. These were termed PC5538III-hc and PC5538III-lc for high and low coverage, respectively. The GC content varied between 27.6% in PC5538III-hc to 28.3% in PC2022III and PC4580III. Most of the genomes assembled nicely, with an average of 31 (8 to 113) contigs per genome. For more detailed assembly statistics, see Table S4 in the supplemental material.

**TABLE 3:** Genome characteristics

Isolate	Genome size (bp)	% GC	No. of:						GenBank accession no.
			Contigs	CDS^*a*^	rRNA	tRNA	Signal peptides	Repeat regions	
Brachyspira aalborgi PC2022III	2,609,344	28.3	15	2,368	3	36	132	1	SAYK00000000
Brachyspira aalborgi PC2777IV	2,655,229	28.1	30	2,449	3	36	142	2	SAYJ00000000
Brachyspira aalborgi PC3053II	2,667,320	28.2	34	2,465	3	36	136	1	SAYI00000000
Brachyspira aalborgi PC3517II	2,645,532	28.2	35	2,430	3	36	138	1	SAYH00000000
Brachyspira aalborgi PC3714II	2,743,156	28.1	24	2,512	3	36	132	1	SAYG00000000
Brachyspira aalborgi PC390II	2,660,153	28.1	32	2,479	3	35	132	1	SAYF00000000
Brachyspira aalborgi PC3939II	2,589,393	28.2	24	2,343	3	36	123	2	SAYE00000000
Brachyspira aalborgi PC3997IV	2,649,511	28.1	33	2,441	3	36	139	1	SAYD00000000
Brachyspira aalborgi PC4226IV	2,715,122	28.1	40	2,549	3	35	137	5	SAYC00000000
Brachyspira aalborgi PC4580III	2,591,031	28.3	10	2,361	3	37	129	1	SAYB00000000
Brachyspira aalborgi PC4597II	2,670,785	28.1	28	2,495	3	35	137	1	SAYA00000000
Brachyspira aalborgi PC5099IV	2,714,460	28.1	21	2,554	3	35	139	2	SAXZ00000000
Brachyspira aalborgi PC5587-p	2,665,010	28.1	33	2,479	3	35	132	1	SAXW00000000
Brachyspira aalborgi PC5587-u	2,662,929	28.1	32	2,483	3	35	133	1	SAXV00000000
Brachyspira pilosicoli PC5538III-hc	2,755,401	27.6	113	2,507	3	34	169	1	SAXY00000000
Brachyspira aalborgi PC5538III-lc	2,746,864	28.1	32	2,500	3	36	139	1	SAXX00000000
Brachyspira aalborgi 513A^T^	2,504,147	28.1	8	2,294	3	36	121	1	SAXU00000000
Brachyspira aalborgi W1	2,666,344	28.2	15	2,413	3	35	128	1	SAXT00000000

a CDS, coding sequences.

**FIG 2:**
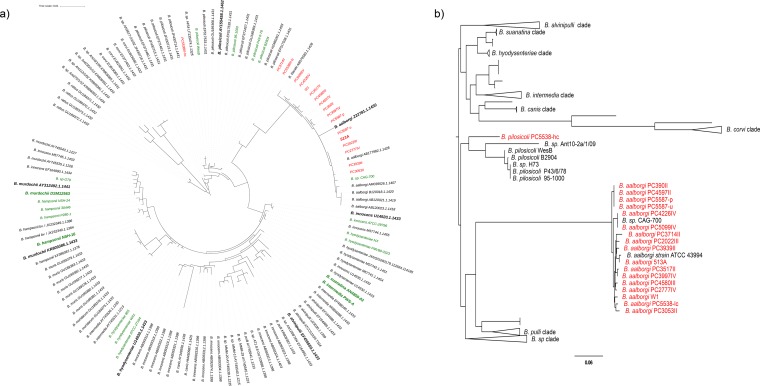
Phylogenetic trees based on the 16S rRNA sequences (a) and the NADH oxidase (NOX) protein sequence (b). The isolates marked in red were sequenced within this study. The sequences marked in green come from the whole-genome sequences described in Table S1. The taxa in bold are the type strains for their respective species. In panel b, clades containing only sequences from one species are collapsed for better readability.

To identify the species of the isolates, rRNA sequences were extracted and searched against the SILVA rRNA database, identifying PC5538III-hc to be Brachyspira pilosicoli and all other isolates to belong to Brachyspira aalborgi (Fig. 2a). Another approach commonly used for the phylogenetic comparison between *Brachyspira* strains is to use the NADH oxidase (*nox*) gene (21). We therefore combined the publicly available *Brachyspira nox* sequences (Table S3) with the *nox* sequences from our strains. The phylogenetic tree can be seen in Fig. 2b and confirms the classification of all but one of the strains to B. aalborgi, grouping together with the previously sequenced *nox* gene of B. aalborgi ATCC 43994 (513A^T^) and the last, PC5538III-hc, to B. pilosicoli.

To investigate the diversity between the *Brachyspira* isolates, we also compared the genomes to publicly available whole-genome sequences within the *Brachyspira* genus, using both average nucleotide identity (ANI) (Fig. S1A), digital DNA-DNA hybridization (dDDH) (Fig. S1B and Table S6), based on gene content in a pangenome analysis (Fig. S1C), and a core genome phylogenetic tree (Fig. S1D), confirming the species identification. Also, we could confirm the previously unclassified *Brachyspira* sp. strain CAG-700, derived from a human gut metagenome (22) to belong to the B. aalborgi species and it was therefore included in further comparative analyses of the species. We could also confirm the recently reported *Brachyspira* sp. strain Z12 (suggested Brachyspira catarrhinii sp. nov.), isolated from a vervet monkey (7), to be divergent from B. aalborgi, with a genome-to-genome distance of, on average, 29.6% (29.5 to 29.7) to the 17 B. aalborgi genomes. Additionally, the other whole-genome-based analyses also supported it being clearly separated from the other species (Fig. S1A to D).

**FIG 3:**
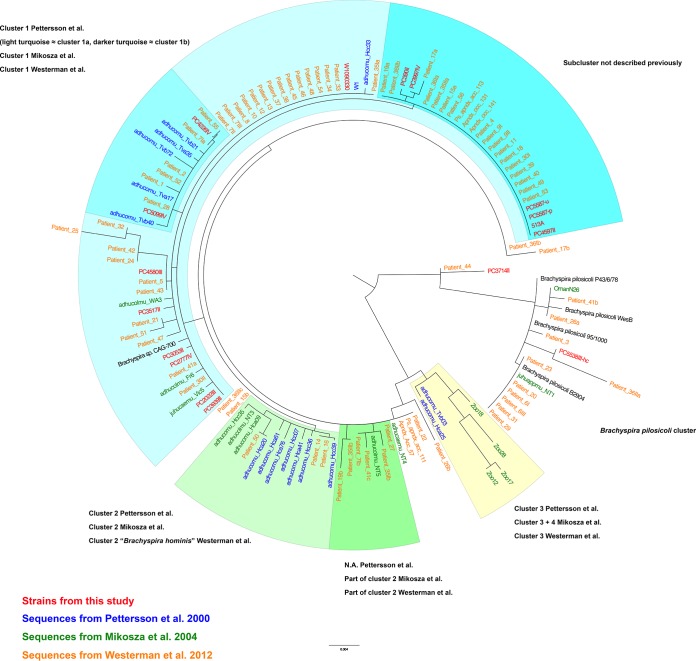
Maximum likelihood tree of 16S rRNA sequences from human intestinal spirochetosis. Isolates in red were sequenced in this study, the ones in blue are from the work of Pettersson et al. (8), isolates in green are from the work of Mikosza et al. (9), and those in orange are from the work of Westerman et al. (10). The phylogenetic clusters are annotated according to the terminology used in the three respective papers.

Since there are no previous studies reporting whole-genome sequences of isolates from human colonic spirochetosis, we made a comparison on the 16S rRNA level with published rRNA sequences from three previous studies on human intestinal spirochetosis: those of Pettersson et al. (8), Mikosza et al. (9), and Westerman et al. (10) (Table S2). The analysis showed that all but two of our CS isolates grouped within previously described cluster 1 (8, 9) (Fig. 3), while isolate PC3714II grouped away from the others, forming its own branch between B. aalborgi and B. pilosicoli, and PC5538III-hc grouped with the B. pilosicoli cluster. None of the isolates grouped within cluster 2 (8, 9) or more specifically within the clade proposed to be called Brachyspira hominis (10) (Fig. 3). Comparison to the results from the biochemical analyses showed no obvious correlation with the phylogenetic patterns.

### Isolates of Brachyspira aalborgi are highly divergent.

Closer investigation and comparative genomics of the 15 B. aalborgi CS genomes show a relatively high heterogeneity between isolates. The ANI ranged between 97.07% and 99.93% (average, 97.49%), where the average percentage of the genomes that aligned in the different comparisons was 86.09% (76.4 to 100%) (see Fig. S1A and Table S6). This could be compared with the average ANI between the 4 sequences available from B. pilosicoli isolates (98.14 to 98.82%; average, 98.32%) and for the 24 publicly available whole-genome-sequenced B. hyodysenteriae isolates (98.92 to 99.96%; average, 99.13%). Also, the dDDH values between the B. aalborgi strains were sometimes below the 70% that is the suggested threshold for a species, on average, 73.6% (68.4 to 99.6%). The PC3714II isolate, which grouped away from the other B. aalborgi isolates in the 16S analysis, did not show any deviant characteristics based on the *nox* analysis, ANI, or dDDH.

### Genome content and comparative genomics.

**FIG 4:**
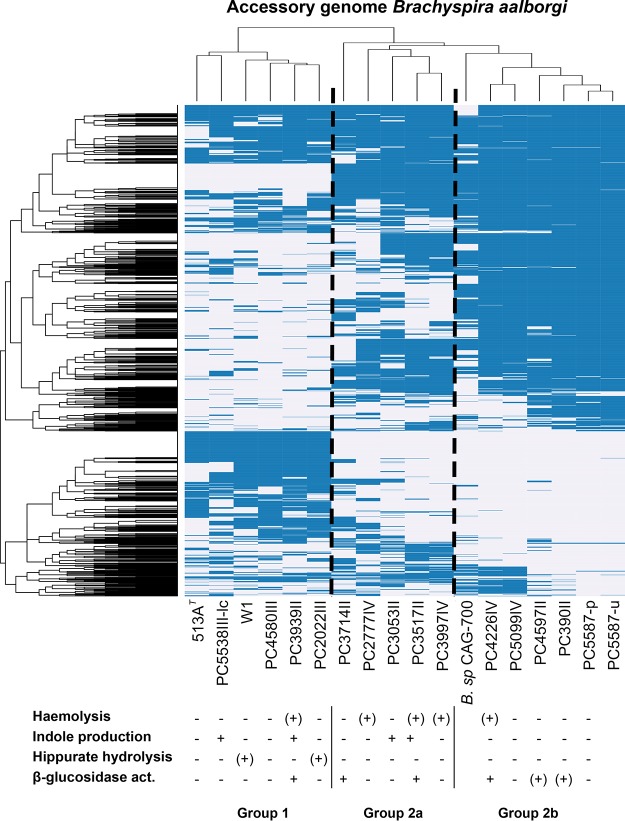
Heat map showing hierarchical clustering of the accessory genome in Brachyspira aalborgi, i.e., genes present in <99% of the genomes, together with the results from the biochemical characterization of isolates. The presence or absence of an orthologous group is shown in blue or white, respectively. Included genomes are those of the 16 newly sequenced B. aalborgi isolates together with *Brachyspira* sp. CAG-700.

Annotation of the new genomes showed a predicted number of coding sequences (CDS) between 2,294 and 2,554 per genome (Table 3). Out of these, on average 49% (46 to 52%) of the predicted CDS were annotated as “hypothetical protein” where no database hit of blast E value <1e−9 could be found in the databases used by the annotation pipeline, i.e., UniProtKB and the HAMAP (23) family profiles. To investigate the difference in functional potential between the isolates, we performed pangenome analysis using the Roary tool. The core genome size (number of genes present in >95% of the isolates) among the B. aalborgi isolates was 1,498 out of the total pangenome of 4,691 genes. This also revealed a large heterogeneity between strains, with three clear clusters of strains with different gene content (Fig. 4).

We also performed a pangenome analysis including the newly sequenced genomes from this study and previously described genomes of the *Brachyspira* genus (see Table S1). Using an amino acid identity cutoff of 70%, we identified 669 core genes that were common for over 99% of the genomes included in the comparison, out of a total pangenome of 8,852 gene clusters. Out of the accessory (noncore) genes, 1,465 were unique for strains of B. aalborgi, out of which 106 were common for all the 18 B. aalborgi genomes. In the same comparison, 982 genes were found only in B. pilosicoli strains, out of which 412 were common for all the 5 B. pilosicoli genomes included in the comparison.

Tools to identify bacterial secretion systems showed all isolates to have genes encoding complete flagellum systems but no other complete secretion systems. We also screened for potential CRISPR-Cas systems, but none was detected. Using the prophage prediction tool ProPhet revealed no putative prophage sequences in any of our newly sequenced genomes.

### Host interaction factors.

Further scrutiny of the B. aalborgi pangenome revealed several genes encoding sialidase, with different numbers of genes in the different strains. Most extreme were strain 513A^T^, in which no sialidase gene was annotated, and PC3714II and PC4226IV, which harbored 13 genes encoding sialidase. Most of the predicted sialidase genes were annotated as sialidase *nedA* by homology to the sequence with UniProtKB accession number Q02834, a sialidase from Micromonospora viridifaciens, but also to the sequence with UniProtKB accession number P15698, a sialidase from Paeniclostridium sordellii, and the sialidase encoded by *nanH*, UniProtKB accession number P10481, from Clostridium perfringens (Fig. S2). Interestingly, many of these genes were unique for B. aalborgi in the pangenome analysis described above.

We also identified another gene encoding a potential mucin-degrading enzyme in the core genome, namely, the protease YdcP, which by Clusters of Orthologous Groups (COG) was classified as a collagenase-like protease, PrtC family. This gene was present in all other *Brachyspira* genomes studied. Also present in all the *Brachyspira* genomes studied was another noteworthy core gene in B. aalborgi, encoding TolC, an outer membrane protein required for the export of virulence proteins and toxic compounds without a periplasmic intermediate (24).

### Spirochetosis is not detected using standard primers for GI tract microbiota analysis.

To verify the presence of *Brachyspira* bacteria in the colonic microbiota using molecular techniques, we used 16S amplicon sequencing where the 16S rRNA was amplified from DNA extracted from the sigmoid biopsy specimens. The method and primer combination used are among the most commonly used to study the human gastrointestinal (GI) microbiota and amplifies the V3-V4 region of the 16S rRNA. In examining the classification of the reads, it was noteworthy that the level of operational taxonomic units (OTUs) classified to the *Brachyspira* genus and even to the *Spirochaetes* phylum was very low (0 to 6.6% of the reads; average, 0.7%) in the individuals with CS and completely undetected in the age- and sex-matched controls (Fig. S3).

To investigate the amplification capacity, we first looked into the potential for primer annealing bioinformatically and observed that the reverse primer had a mismatch in the nondegenerate positions close the 3′ end, which we suspected would lead to poor amplification efficiency (Fig. S4). To verify this experimentally we made mixes of genomic DNA from five of the *Brachyspira* isolates at a fixed DNA-to-DNA ratio (1:3) with a microbial community standard of known composition to evaluate the performance of the primers. The results indicated that 0.05 to 0.7% of the amplified reads were classified as *Brachyspira*, much below the expected 25%. The higher number (0.7%) was seen for B. pilosicoli strain PC5538III (Fig. S5).

Despite the failure of the primers to amplify *Brachyspira* 16S rRNA, we evaluated whether CS status verified by histopathology was associated with alterations in the microbial community composition and diversity. However, this did not reveal any significant differences between the groups, as illustrated by the lack of segregation between the groups using principal-component analysis (Fig. S6A) or in terms of Shannon diversity (*P* = 0.07) or Chao1 (*P* = 0.41) index (Fig. S6B and S6C).

### Looking for CS in the Human Microbiome Project data.

To look for spirochetosis in a publicly available data set from another population-based microbiota study, we downloaded the 16S amplicon data from stool samples from 324 individuals from the Human Microbiome Project (HMP) (23). These samples had been amplified using two different primer combinations compared to our data set, one amplifying the V1-V3 region of the 16S rRNA and the other the V3-V5 region. We also downloaded assembled shotgun metagenome sequences from 179 individuals to see if we could find any signs of *Brachyspira* sequences in those data. We found two shotgun metagenome assemblies containing *Brachyspira* bacteria, and those two samples also had 16S amplicon reads classified to the *Brachyspira* genus; however, the levels were almost undetectable, 0.03% (V3-V5) and 0.04% (V1-V3) *Brachyspira* reads (Fig. S7). These assemblies came from the same individual, albeit from different stool samples. Further investigation of the primer pairs used for the two amplification protocols used show that, like 341F/805R, both of these primer combinations are likely to be very inefficient in amplifying *Brachyspira* 16S rRNA due to primer mismatches (Fig. S4).

## DISCUSSION

Human colonic spirochetosis (CS) is a condition of striking histological appearance, with bacteria adhering to the colonic epithelium (1, 12, 25). Despite this, the clinical impact of CS is not established, and successful culture of spirochetes from human material is rarely reported. Nor have any isolates from human CS been characterized at the whole-genome level. Here we report isolates and whole-genome sequences from 14 individuals with CS, together with the Brachyspira aalborgi type strain, 513A (ATCC 43994), and previously described clinical isolate W1 (13).

Out of the 17 individuals with histological CS, we failed to isolate spirochete colonies from 3. Interestingly, all three of these individuals were diagnosed with IBS based on Rome III criteria, and both of the individuals with IBS diarrhea were among these. In total we obtained viable isolates from only 3 out of the 6 individuals with CS and IBS. Comparing the 16S rRNA sequences of the sequenced isolates with previously published studies of samples from human CS, isolates belonging to clusters 2 to 4 in the phylogenetic tree are missing in our material. This could be because none of the individuals in our study carried these kinds of bacteria but could also be because only cluster 1 isolates are cultivable, as assumed by others (9). However, isolates in cluster 1 also seem to be more commonly found when other methods are used (26).

In trying to circumscribe the species of the isolates, we were limited by the fact that no B. aalborgi whole-genome sequences were hitherto available, and we therefore also included the B. aalborgi type strain, 513A, in the sequencing. We then compared the 16S rRNA sequences of the newly sequenced isolates to publicly available *Brachyspira* 16S sequences from the SILVA database and delineated the phylogeny based on the *nox* gene (Fig. 2) (21). This allowed us to classify 14 of the patient isolates as Brachyspira aalborgi and one as Brachyspira pilosicoli, which was further confirmed with better resolution using ANI and dDDH comparison to publicly available *Brachyspira* whole-genome sequences (Fig. S1).

Using the comparative metrics described above, we could also see that the sequence diversity within B. aalborgi is high compared to that in the other *Brachyspira* species for which several whole-genome sequences are available, despite the fact that the CS isolates were all from a limited geographical area consisting of two adjacent parishes in Stockholm, Sweden. The proposed and generally accepted species boundaries for ANI and dDDH values are 95 to 96% and 70%, respectively (27) and the lowest ANI value within the *aalborgi* genomes was 97.07%. However, the lowest dDDH value was 68.4%, and several strains showed this level of similarity to the type strain. This means that several isolates are on the borderline of qualifying as novel species, but there is also a noteworthy continuum of decreasing similarity values, and more isolates from human colonic spirochetosis would be needed to correctly delineate further differentiation within the *Brachyspira* genus. Also, the biochemical tests did not show any correlation with the ANI, dDDH, or accessory genome-based groupings of the isolates, suggesting that these tests, which have been proven useful in characterization of several other *Brachyspira* species, cannot be used for species designation of B. aalborgi isolates. A high sequence diversity and signs of extensive recombination have also been shown for B. pilosicoli using both multilocus enzyme electrophoresis (MLEE) (28) and MLST (29), and for whole-genome comparisons of the four previously sequenced B. pilosicoli isolates (14), but with the whole-genome sequences available to date, this variation seems to be lower than within the B. aalborgi species. It is noteworthy also that the B. pilosicoli sequences included in the comparison are from diverse geographical areas and also from different host species (14), while the B. aalborgi genomes are all from human infections and from a relatively limited geographical area of low socioeconomic diversity in one developed country, with the exception of the reference strains that were isolated in Denmark and an adjacent part of Sweden. Since colonic spirochetosis is more prevalent in developing areas in the world (30), this sample is very likely just scratching the surface of the total global diversity of bacteria from human colonic spirochetosis.

To investigate whether the genomes encoded proteins that could participate in host-bacterium interaction and/or explain the capacity of the spirochetes to penetrate both the loose and inner, attached mucus layers in the colon, we searched the genomic content of the CS isolates for genes involved in mucus metabolism. We found an abundance of sialidases in the B. aalborgi genomes, ranging from 2 to 13 genes encoding sialidases per isolate, with the one exception being the type strain, 513A, which interestingly did not harbor any sialidase genes. Sialidases are proteins that hydrolyze α2,3-, α2,6-, and α2,8-glycosidic linkages of terminal sialic acid residues in oligosaccharides, glycoproteins, and glycolipids (31). Sialidated sugars are abundant in mucins and other glycoproteins on the intestinal epithelium, where there is a gradient of increasing terminal sialic acids from the ileum to the rectum in humans (32). The purpose of this cleavage can be to release sialic acids for use as carbon and energy sources, whereas in pathogenic microorganisms, sialidases have been suggested to be pathogenic factors where the bacteria use the sialic acid to evade the immune system (33). Sialidase/sulfatase genes have been previously been found in B. pilosicoli and B. aalborgi but not in B. hyodysenteriae (34). Interestingly, the large majority of the sialidase genes identified in the B. aalborgi genomes did not have homologues in the other *Brachyspira* species according to the pangenome analysis. However, some were shared with the *Brachyspira* sp. Z12, which was previously misclassified as B. aalborgi but now has been proposed to belong to a novel species, B. catarrhinii (7). This multitude of highly homologous genes also posed problems for the *de novo* assembly, and several of the annotated sialidase genes were truncated due to contig breaks.

Another potential virulence factor in B. aalborgi is the collagenase PrtC. This protease was originally described for Porphyromonas gingivalis and has also been found in other human pathogens, such as Helicobacter pylori, Salmonella enterica, Escherichia coli, and Bacillus subtilis (35). Bacterial collagenases have been highlighted in pathogenicity due to their ability to cleave extracellular matrix components and thereby facilitate colonization and invasion (36), and PrtC has also been suggested to act as an adhesin to collagenous structures (37).

Another gene encoding what seems to be a core protein in *Brachyspira* is the TolC gene, located between the genes encoding the AcrAB multidrug efflux pump system subunits AcrB and AcrA (membrane fusion protein) (38). The AcrAB-TolC efflux pump, which spans the inner and outer membranes of the bacterium, is able to transport a broad range of structurally unrelated small molecules/drugs out of certain Gram-negative bacteria, and this AcrAB-TolC complex has been shown to confer antibiotic resistance and survival in the gastrointestinal tract (39) and also has been described for B. pilosicoli (14).

Detection of spirochetosis by histology requires invasive methods such as colonoscopy and collection of biopsied for histology assessment. To see if the spirochetes could be detected by standard 16S amplicon studies of the microbiota, we performed such an analysis using a primer combination commonly used in population-based studies of the human microbiota. To our surprise, the level of reads classified in the *Brachyspira* genus was very low despite being amplified from tissue locations adjacent to those where an abundance of spirochetes could be observed under a microscope. To identify the reason for this detection failure, we compared the primers used in our study and the HMP study, from which we also analyzed samples, and which is also a large population-based study. The results are shown in Fig. S4 in the supplemental material and verified that none of the three primer combinations could efficiently amplify *Brachyspira* 16S rRNA in our study due to a mismatch in the 805R primer. The HMP studies either used V3-V5 357F plus 926R or V1-V3 27F plus 534R (40), for which V1-V3 amplification of *Brachyspira* is hampered due to a mismatch in 27F and for the V3-V5 amplification a mismatch in 926R. Considering that spirochetosis based on observations in the PopCol study increase the risk for having IBS 3-fold (17), this failure to detect *Brachyspira* using standard protocols for microbiota studies should be taken seriously since this potentially important etiological agent in the pathology of IBS will be missed.

The current study had a number of strengths, being population based with good representation of the general population in the investigated areas, and the distribution of most relevant socioeconomic parameters was similar to Swedish national averages (20). There were also weaknesses. We obtained viable isolates from only a few individuals with IBS, and considering this together with the large genetic heterogeneity between isolates, we did not have statistical power to further investigate associations between bacterial genetic traits and lower gastrointestinal symptoms, including inflammatory parameters such as eosinophil infiltration. However, the notion of the poor success rate in culturing IBS isolates could also be important and should be investigated further. Also, studying more isolates and genomic sequences in the future from human CS in general would allow us to make more thorough inferences on functional genomics and pathogenic potential. Although the spirochetes had similar morphologies in tissue sections, we observed differences in phenotype—cell size and shape and growth rate—when culturing the isolates. Previous studies have shown that there might exist a considerable complexity in the spirochete population in each individual (8), but our approach of purifying and sequencing one isolate per individual did not allow us to investigate this. However, from one of the individuals we had two morphologically different isolates identified, and from one, the genome sequences turned out to consist of two species, suggesting that there is more to be investigated in this respect.

In conclusion, we report for the first time the whole-genome analysis of a collection of human clinical isolates from individuals with colonic spirochetosis, showing that genomic diversity between isolates is high. From one individual we could retrieve whole-genome sequences from two distinct species, confirming the observation of mixed infections. The sequencing of the B. aalborgi type strain also allowed us to make genome-based species circumscription with high resolution compared to previous 16S and *nox*-based investigations. We found several genes in the spirochetosis genomes that suggest intimate host-microbe interaction and that could confer a pathogenic potential to spirochetes in the human colon, such as a multitude of genes encoding mucin-degrading proteins. The association of these bacteria with, for example, IBS and the notion that they go undetected in major studies of the gut microbiota raise questions as to whether they represent an underestimated etiological agent in functional bowel disorders, an issue that should be considered when designing future microbiota analyses.

## MATERIALS AND METHODS

### Population.

The population-based colonoscopy (PopCol) study has been described in detail elsewhere (20). Briefly, random samples of the adult population in two adjacent parishes in Södermalm, Stockholm, were sent a validated abdominal symptom questionnaire (ASQ) (41). Of 3,556 recipients, 2,293 responded to the questionnaire; of these, 1,643 persons were reached by phone with an invitation to an interview with a colonoscopist and to have a subsequent colonoscopy. A total of 1,244 participated in the interview; of these, 745 also underwent colonoscopy. The subjects who underwent colonoscopy were similar to the total population enrolled (20). For 17 of the individuals spirochetes were observed in hematoxylin-and-eosin-stained tissue sections, which were subsequently confirmed by immunohistochemistry (IHC) using a polyclonal rabbit antiserum (13, 17). The study was approved by the local ethics committee (no. 394/01; Forskningskommité Syd), and all participants gave their informed consent. Irritable bowel syndrome (IBS) and bowel habit subtypes (diarrhea, mixed diarrhea and constipation, and unclassifiable) were defined by applying Rome III criteria (42) and have been described in detail by Walker et al. (17).

### Collection of biopsy specimens.

The colonoscopies were performed by 7 experienced endoscopists after cleansing of the bowel using Phosphoral (Recordati AB, Sweden). Biopsy specimens were taken at 4 levels in the colon (cecum, transverse, sigmoid, and rectum) and in the terminal ileum, and the biopsied sites were chosen randomly at each level. For more details of biopsy specimen collection, see the work of Kjellström et al. (20).

For both the isolation of bacteria and the 16S amplicon sequencing, sigmoid colon biopsy specimens were used. For the microbiota analysis, four age- and sex-matched controls without spirochetosis per CS case were randomly selected from the participants with normal pathology (both macroscopic and microscopic) from the PopCol study.

### Isolation and biochemical characterization of spirochetes.

Culturing of the spirochetes was performed from biopsy specimens that had been stored in freezing medium consisting of Tris-buffered saline with 10% glycerol at pH 7.0. This was done after the immunohistochemical confirmation of the CS cases, 1 year after the colonoscopy study was completed. Biopsy specimens from the 17 individuals with confirmed spirochetosis were homogenized in the freezing medium, and 50 μl was cultivated anaerobically on selective medium for spirochete isolation and subculturing as previously described (13). In brief, a selective medium was used, consisting of tryptose soy agar (TSA) to which were added 10% bovine blood, 400 μg of spectinomycin per ml, and 5 μg of polymyxin per ml. Pure growth of spirochetal bacteria was confirmed with a phase-contrast microscope. From three of the individuals we could not isolate any spirochetes by our methods. From one individual two isolates were selected since they displayed different morphologies on the plate. B. aalborgi strain 513A^T^ (ATCC 43994/NCTC 11492) (12) and the Swedish clinical B. aalborgi isolate W1 (13), from a strain collection at the National Veterinary Institute, Uppsala, were also cultured and used for comparison. See Table 1 for a summary of the individuals and isolates.

The spirochetes were tested for indole production, their ability to hydrolyze sodium hippurate, beta-hemolysis capacity, and cellular α-galactosidase and β-glucosidase activities, as has been described previously (68).

### DNA extraction and sequencing library preparation of spirochetes.

The spirochetes were harvested and DNA was extracted from the pellet using the DNeasy blood and tissue kit (Qiagen, Hilden, Germany) with proteinase K treatment at 56°C for enhanced lysis.

Library preparation was performed using the TruSeq Nano DNA library preparation kit (Illumina) using the 550-bp insert size protocol and the libraries were subsequently sequenced on the MiSeq platform, v2 chemistry, 300-bp paired-end reads. The average coverage of the genomes was >300-fold and the average insert size 900 bp. For more detailed sequencing and assembly statistics, see Table S1.

### Bioinformatics analysis of whole-genome sequences. (i) *De novo* genome assembly and annotation.

Reads were quality assessed and trimmed using bbduk (bbmap v. 37.77) (43) and *de novo* assembled using SPAdes v 3.11.1 (44) assembly, using the –careful option and kmer options 21, 33, 55, 77, 99, and 127, and the assemblies were filtered to remove contigs of low coverage or a length under 500 bp. The draft genome of strain 513A^T^ was ordered using the Mauve (45) Order Contig function to the incomplete draft of Brachyspira aalborgi 513A^T^ available at the MetaHIT consortium repository (https://www.sanger.ac.uk/resources/downloads/bacteria/metahit/), and the other draft genomes were subsequently ordered to the 513A^T^ draft. The ordered drafts were annotated using the *prokka* annotation pipeline v. 1.12 (46).

### (ii) Species circumscription and phylogeny.

To investigate the species designation of the CS isolates, phylogenetic analysis of 16S rRNA sequences was performed by comparing the 16S sequences of the human CS strains to the Ref and RefNR *Brachyspira* 16S sequences retrieved from the SILVA database (https://www.arb-silva.de/). The sequences were aligned using muscle (47), and a maximum likelihood tree was constructed using PhyML (40), both software integrated in Seaview v. 4.5.4 (48).

The NADH oxidase gene (*nox*) has often been used for phylogenetic comparison of spirochetes (21) and was analyzed by combining publicly available *Brachyspira nox* sequences from GenBank (see Table S3 in the supplemental material) and the *nox* sequences from our newly sequenced strains. The nucleotide sequences were aligned using muscle and a phylogenetic tree was reconstructed using PhyML as described above.

To study the genetic variability between the CS strains and hitherto described genomes within the *Brachyspira* genus listed in Table S1, an average nucleotide identity (ANI) analysis was performed using MUMmer alignment (49) implemented in *pyani* software v. 0.2.7 (https://github.com/widdowquinn/pyani).

*Brachyspira* sp. strain CAG-484 (GCA_000431315.1), a metagenomic assembly from the metaHIT Consortium, was excluded from these analyses since it was highly divergent from the others and seemed to be misclassified to the *Brachyspira* genus.

Digital DNA-DNA hybridization (dDDH) analysis was performed using the Genome-to-Genome Distance Calculator (GGDC) web tool v. 2.1 (50).

To compare the genetic contents of the isolates, the Roary pangenome pipeline (51) was used, with an identity cutoff of 70% on protein level. This analysis was performed both including all the above-mentioned *Brachyspira* genome references and then separately for B. pilosicoli (PC5538III-hc and the four publicly available genomes) and for the B. aalborgi genomes. To construct a core genome phylogeny, PhyML software v. 3.1 (40) was applied to the core genome alignment generated by Roary. The presence or absence of genes in the resulting accessory genome was clustered using the pvclust package (52).

A more detailed analysis of strains associated with human colonic spirochetosis was also performed by retrieving the 16S sequences described by Mikosza et al. (9), Pettersson et al. (8), and Westerman et al. (10) (Table S2). The sequences were aligned and a PhyML tree was constructed as described above.

### (iii) Comparative genomics and functional genomics analysis.

To further investigate the functional characteristics of the genomes, we further scrutinized the Roary pangenome data by classifying the representative sequences of each core and accessory gene cluster according to KEGG orthology using the BlastKOALA online tool (53). We also classified all B. aalborgi strain 513A^T^ genes into Clusters of Orthologous Groups (COG) categories (54) to facilitate functional interpretation. To further functionally characterize the genomes we applied the macsyfinder *TXSScan* tool (55) to identify bacterial secretion systems (type I to type VI secretion systems [T1SS to T6SS], T9SS, flagella, type IV pili, and Tad pili) and the prophage prediction tool ProPhet (56).

### Colonic microbiota composition in individuals with CS compared to controls. (i) DNA extraction.

The homogenized sigmoid colon biopsy specimens were transferred from their original freezing medium into DNA/RNA Shield lysis buffer prior to extraction with ZymoBIOMICS (Zymo Research Corp., Irvine, CA).

The biopsy specimens were subjected to bead beating with Matrix E (MP Biomedicals, Santa Ana, CA) in a 96 FastPrep shaker (MP Biomedicals) for 2 to 6 min (the beating proceeds until the sample is visually homogeneous), and samples were then spun down to remove beads from the solution. The supernatants were then incubated in lysozyme buffer (20 mM Tris-Cl, 2 mM sodium EDTA, lysozyme to 100 g/ml; Sigma, St. Louis, MO) at 37°C for 45 min to 1 h at 1,000 rpm. Following this, samples were again spun down and the supernatants were transferred to a new plate, to eliminate larger particles, and then incubated with proteinase K at 55°C at 250 rpm for 30 min. Finally, the samples were cleaned through several washing and magnetic bead pelleting steps according to the instructions of the manufacturer (Genomic DNA MagPrep kit; Zymo Research Corp., Irvine, CA) and the DNA was eluted from the magnetic beads with 70 μl of elution buffer (10 mM Tris-Cl, pH 8.5; Qiagen, Venlo, Netherlands). The DNA extraction was automated on the FreedomEVO robot (TECAN Trading AG, Männendorf, Switzerland).

A blank negative control and a positive mock control (ZymoBIOMICS mock community standard; Zymo Research Corp.) were included in each extraction round.

### (ii) DNA amplification and sequencing.

Prior to amplification, samples were normalized and a total of 170 ng of DNA was used to amplify the V3-V4 region of the 16S rRNA gene using primer pair 341F/805R (57).

For the 1-step PCR procedure, amplification was carried out by a high-fidelity proofreading polymerase for a total of 25 cycles. For amplification of the sequencing libraries, forward primer 5′-CAAGCAGAAGACGGCATACGAGAT-N_8_-GTCTCGTGGGCTCGGAGATGTGTATAAGAGACAGGACTACHVGGGTATCTAATCC-3′ and reverse primer 5′-AATGATACGGCGACCACCGAGATC-N_8_-TCGTCGGCAGCGTCAGATGTGTATAAGAGACAGCCTACGGGNGGCWGCAG-3′, where N_8_ represents an identifying 8-mer (barcode) and the last 21 and 19 bases in each construct are the sequence-specific forward and reverse primers, respectively, were used.

Samples were then pooled to equimolar amounts and sequenced in parallel to whole bacterial genomes on the MiSeq instrument (Illumina Inc., San Diego, CA). All controls from the extraction phase, as well as a negative (blank) PCR control, were also prepared and sequenced.

### (iii) Sequence correction and taxonomic assignment.

The processing of 16S amplicon reads was performed as described previously (58). Briefly, Cutadapt (59) was used to eliminate all sequences not containing the amplification primers, remove the primer sequences, all bases with a Phred score below 15, and all reads with less than 120 bp left after trimming. The resulting reads were merged with usearch v. 9.0.2132 (60), and reads failing to merge or producing merging products shorter than 380 bp, longer than 520 bp, or with more than three expected errors were discarded. All unique full-length samples occurring at a frequency higher than 10^−6^ in the data set were submitted to unoise (61). All the merged reads were then mapped back to the accepted centroids and assigned to the OTU with the highest identity, at a minimum of 98%. In cases of equally high identity matches, the most abundant centroid was selected. Taxonomy was assigned using SINA v. 1.2.13 (62). Scripts for performing this analysis are available at https://github.com/ctmrbio/Amplicon_workflows.

### (iv) Statistical analysis.

For statistical analyses, all samples with less than 5,000 total OTUs were discarded, as were OTUs not present in at least 3 samples. The resulting filtered OTU counts were parsed to group counts on the different taxonomical levels and visualized using the R software packages ggplot (63) and pheatmap (64). Shannon and Chao1 alpha diversity statistics and Bray-Curtis similarity were calculated using the vegan R package (65). Differences in diversity statistics between spirochetosis cases and controls were tested using Student’s *t* test.

### (v) Verification of 16S primer performance.

Due to the low total counts of reads from the biopsy specimen amplicon sequencing assigned to the *Brachyspira* genus, we performed a control experiment to verify the amplification performance of the 341F/805R primer combination on mixes of bacterial DNA. The mixes were prepared of 75% ZymoBIOMICS microbial community DNA standard and 25% DNA from five of the different *Brachyspira* isolates, selected from different clades of the 16S rRNA phylogenetic tree (Fig. 2), namely, B. aalborgi 513A^T^, W1, PC3939II, and PC3714II and B. pilosicoli PC5538III. The standard is a defined mixture of 5 Gram-positive and 3 Gram-negative bacteria plus 2 yeast species with wide GC range (15% to 85%) for evaluation and optimization of microbiomics workflows. A pure ZymoBIOMICS microbial community DNA standard sample and a blank sample were also included as positive and negative PCR controls.

### (vi) Analysis of Human Microbiome Project data.

To search for the presence of spirochetes in the publicly available microbiota data from the Human Microbiome Project (HMP), we downloaded 16S amplicon data from 324 stool samples and metagenomic assemblies from 179 stool samples (Table S5). The 16S samples were analyzed with the methods described above, while the assembled shotgun metagenome data were screened for the presence of spirochetes using MASH (66). Briefly, the Brachyspira aalborgi and B. pilosicoli genomes previously described in this study were combined and a MASH sketch was created from these, to which all the downloaded assemblies were compared. The assemblies showing signs of containing spirochete sequences were aligned to the combined genomes using blastn (67), and the contigs having hits with an E value of lower than e−9 were extracted for further investigation.

### Data availability.

The genomes are submitted to GenBank under BioProject no. PRJNA513011.

## Supplementary Material

Supplemental file 1
