# Supplementary material for: Isolates from Colonic Spirochetosis in Humans Show High Genomic Divergence and Potential Pathogenic Features but Are Not Detected Using Standard Primers for the Human Microbiota
Source: J Bacteriol. 2019 Oct 4;201(21):e00272-19. doi: 10.1128/JB.00272-19 (PMC6779451; doi:10.1128/JB.00272-19)
Supplement: Supplemental file 1 [file JB.00272-19-s0001.pdf]

Supplementary table 1. Reference *Brachyspira* sequences

Assembly accession	Species	Strain	Assembly level	Size (Mb)	GC%	FTP path
GCA_000518245.1	*Brachyspira alvinipulli*	ATCC 51933	Scaffold	3.421	26.9	ftp://ftp.ncbi.nlm.nih.gov/genomes/all/GCA/000/518/245/GCA_000518245.1_ASM51824v1
GCA_000316195.1	*Brachyspira hampsonii*	30446	Scaffold	3.037	27.6	ftp://ftp.ncbi.nlm.nih.gov/genomes/all/GCA/000/316/195/GCA_000316195.1_ASM31619v1
GCA_000334935.1	*Brachyspira hampsonii*	30599	Contig	2.943	27.8	ftp://ftp.ncbi.nlm.nih.gov/genomes/all/GCA/000/334/935/GCA_000334935.1_brachy30599_1.0
GCA_001746225.1	*Brachyspira hampsonii*	NSH-16	Contig	3.161	27.4	ftp://ftp.ncbi.nlm.nih.gov/genomes/all/GCA/001/746/225/GCA_001746225.1_ASM174622v1
GCA_001746215.1	*Brachyspira hampsonii*	NSH-24	Contig	2.969	27.5	ftp://ftp.ncbi.nlm.nih.gov/genomes/all/GCA/001/746/215/GCA_001746215.1_ASM174621v1
GCA_001746205.1	*Brachyspira hampsonii*	P280/1	Contig	3.187	27.5	ftp://ftp.ncbi.nlm.nih.gov/genomes/all/GCA/001/746/205/GCA_001746205.1_ASM174620v1
GCF_000022105.1	*Brachyspira hyodysenteriae*	WA1; ATCC 49526	Complete Genome	3.037	27	ftp://ftp.ncbi.nlm.nih.gov/genomes/all/GCF_000022105.1_ASM2210v1
GCA_001012935.1	*Brachyspira hyodysenteriae*	865	Scaffold	3.010	27	ftp://ftp.ncbi.nlm.nih.gov/genomes/all/GCF_001012935.1_ASM101293v1
GCF_000383255.1	*Brachyspira hyodysenteriae*	ATCC 27164, B78	Scaffold	3.050	27.1	ftp://ftp.ncbi.nlm.nih.gov/genomes/all/GCF_000383255.1_ASM38325v1
GCA_001012915.1	*Brachyspira hyodysenteriae*	FMV89.3323	Scaffold	3.167	27	ftp://ftp.ncbi.nlm.nih.gov/genomes/all/GCA/001/012/915/GCA_001012915.1_ASM101291v1
GCF_001012925.1	*Brachyspira hyodysenteriae*	NX	Scaffold	3.014	27	ftp://ftp.ncbi.nlm.nih.gov/genomes/all/GCA/001/012/925/GCA_001012925.1_ASM101292v1
GCF_000384655.1	*Brachyspira innocens*	ATCC 29796, B256	Contig	3.282	27.7	ftp://ftp.ncbi.nlm.nih.gov/genomes/all/GCF_000384655.1_ASM38465v1
GCF_000223215.1	*Brachyspira intermedia*	PWS/A	Complete Genome	3.308	27.2	ftp://ftp.ncbi.nlm.nih.gov/genomes/all/GCF_000223215.1_ASM22321v1
GCF_000092845.1	*Brachyspira murdochii*	DSM 12563	Complete Genome	3.242	27.8	ftp://ftp.ncbi.nlm.nih.gov/genomes/all/GCF_000092845.1_ASM9284v1
GCF_000143725.1	*Brachyspira pilosicoli*	95/1000	Complete Genome	2.586	27.9	ftp://ftp.ncbi.nlm.nih.gov/genomes/all/GCF_000143725.1_ASM14372v1
GCF_000296575.1	*Brachyspira pilosicoli*	B2904	Complete Genome	2.765	27.8	ftp://ftp.ncbi.nlm.nih.gov/genomes/all/GCF_000296575.1_ASM29657v1
GCF_000325665.1	*Brachyspira pilosicoli*	P43/6/78	Complete Genome	2.556	27.9	ftp://ftp.ncbi.nlm.nih.gov/genomes/all/GCF_000325665.1_ASM32566v1
GCF_000319185.1	*Brachyspira pilosicol*	WesB	Chromosome	2.890	27.9	ftp://ftp.ncbi.nlm.nih.gov/genomes/all/GCF_000319185.1_ASM31918v1
GCF_001049755.1	*Brachyspira suanatina*	AN4859/03	Contig	3.258	27	ftp://ftp.ncbi.nlm.nih.gov/genomes/all/GCA/001/049/755/GCA_001049755.1_BRSU_AN4859_03
GCA_000431595.1	*Brachyspira sp.*	CAG:700	Scaffold	2.458	28.2	ftp://ftp.ncbi.nlm.nih.gov/genomes/all/GCA/000/431/595/GCA_000431595.1_MGS700
GCA_002379365.1	*Brachyspira sp.*	G79	Scaffold	3.285	29.3	ftp://ftp.ncbi.nlm.nih.gov/genomes/all/GCA/002/379/365/GCA_002379365.1_ASM237936v1

Supplementary table 2. 16S sequences included as references

Accession number	Species	Name	16S type	Reference
gb|AY187061.1	Uncultured *Brachyspira sp*. clone	Zoo28	partial sequence	Mikosza et. al[1]
gb|AY187060.1	Uncultured *Brachyspira sp*. clone	Zoo18	partial sequence	Mikosza et. al[1]
gb|AY187059.1	*Brachyspira sp.*	Zoo17	partial sequence	Mikosza et. al[1]
gb|AY187058.1	*Brachyspira sp.*	Zoo12	partial sequence	Mikosza et. al[1]
gb|AY187057.1	*Brachyspira pilosicoli*	OmanN26	partial sequence	Mikosza et. al[1]
gb|AY187056.1	Uncultured *Brachyspira sp*. clone	juhucaemu_Vic5	partial sequence	Mikosza et. al[1]
gb|AY187055.1	Uncultured *Brachyspira sp*. clone	adhucolmu_WA3	partial sequence	Mikosza et. al[1]
gb|AY187054.1	Uncultured *Brachyspira sp*. clone	adhucolmu_NT5	partial sequence	Mikosza et. al[1]
gb|AY187053.1	Uncultured *Brachyspira sp*. clone	adhucaemu_NT4	partial sequence	Mikosza et. al[1]
gb|AY187052.1	Uncultured *Brachyspira sp*. clone	adhucolmu_NT3	partial sequence	Mikosza et. al[1]
gb|AY187051.1	Uncultured *Brachyspira sp*. clone	juhuappmu_NT1	partial sequence	Mikosza et. al[1]
gb|AY187050.1	Uncultured *Brachyspira sp*. clone	adhucolmu_Fr6	partial sequence	Mikosza et. al[1]
gb|JX446555.1	*Brachyspira pilosicoli* clone	Patient_41b	partial sequence	Westerman et. al[2]
gb|JX446554.1	*Brachyspira aalborgi* clone	Patient_41a	partial sequence	Westerman et. al[2]
gb|JX446548.1	*Brachyspira aalborgi* clone	Patient_36IIb	partial sequence	Westerman et. al[2]
gb|JX446547.1	*Brachyspira pilosicoli* clone	Patient_36IIa	partial sequence	Westerman et. al[2]
gb|JX446545.1	*Brachyspira aalborgi* clone	Patient_36Ia	partial sequence	Westerman et. al[2]
gb|JX446543.1	*Brachyspira aalborgi* clone	Patient_35IIa	partial sequence	Westerman et. al[2]
gb|JX446541.1	*Brachyspira aalborgi* clone	Patient_35Ia	partial sequence	Westerman et. al[2]
gb|JX446530.1	*Brachyspira pilosicoli* clone	Patient_26a	partial sequence	Westerman et. al[2]
gb|JX446522.1	*Brachyspira aalborgi* clone	Patient_19a	partial sequence	Westerman et. al[2]
gb|JX446519.1	*Brachyspira aalborgi* clone	Patient_17a	partial sequence	Westerman et. al[2]
gb|JX446516.1	*Brachyspira aalborgi* clone	Patient_15a	partial sequence	Westerman et. al[2]
gb|JX446504.1	*Brachyspira aalborgi* clone	Patient_7Ia	partial sequence	Westerman et. al[2]
gb|JX446571.1	*Brachyspira aalborgi* clone	Patient_56	partial sequence	Westerman et. al[2]
gb|JX446570.1	*Brachyspira aalborgi* clone	Patient_55	partial sequence	Westerman et. al[2]
gb|JX446569.1	*Brachyspira aalborgi* clone	Patient_54	partial sequence	Westerman et. al[2]
gb|JX446568.1	*Brachyspira aalborgi* clone	Patient_53	partial sequence	Westerman et. al[2]
gb|JX446567.1	*Brachyspira aalborgi* clone	Patient_52	partial sequence	Westerman et. al[2]
gb|JX446566.1	*Brachyspira aalborgi* clone	Patient_51	partial sequence	Westerman et. al[2]
gb|JX446565.1	Uncultured *Brachyspira sp*. clone	Patient_50	partial sequence	Westerman et. al[2]
gb|JX446564.1	*Brachyspira aalborgi* clone	Patient_49	partial sequence	Westerman et. al[2]
gb|JX446563.1	*Brachyspira aalborgi* clone	Patient_48	partial sequence	Westerman et. al[2]
gb|JX446562.1	*Brachyspira aalborgi* clone	Patient_47	partial sequence	Westerman et. al[2]
gb|JX446561.1	*Brachyspira aalborgi* clone	Patient_46	partial sequence	Westerman et. al[2]
gb|JX446560.1	*Brachyspira aalborgi* clone	Patient_45	partial sequence	Westerman et. al[2]
gb|JX446559.1	Uncultured *Brachyspira sp*. clone	Patient_44	partial sequence	Westerman et. al[2]
gb|JX446558.1	*Brachyspira aalborgi* clone	Patient_43	partial sequence	Westerman et. al[2]
gb|JX446557.1	*Brachyspira aalborgi* clone	Patient_42	partial sequence	Westerman et. al[2]
gb|JX446556.1	Uncultured *Brachyspira sp*. clone	Patient_41c	partial sequence	Westerman et. al[2]
gb|JX446553.1	*Brachyspira aalborgi* clone	Patient_40	partial sequence	Westerman et. al[2]
gb|JX446552.1	*Brachyspira aalborgi* clone	Patient_39	partial sequence	Westerman et. al[2]
gb|JX446551.1	*Brachyspira aalborgi* clone	Patient_38	partial sequence	Westerman et. al[2]
gb|JX446550.1	*Brachyspira aalborgi* clone	Patient_37	partial sequence	Westerman et. al[2]
gb|JX446549.1	Uncultured *Brachyspira sp*. clone	Patient_36IIc	partial sequence	Westerman et. al[2]
gb|JX446546.1	Uncultured *Brachyspira sp*. clone	Patient_36Ib	partial sequence	Westerman et. al[2]
gb|JX446544.1	Uncultured *Brachyspira sp*. clone	Patient_35IIb	partial sequence	Westerman et. al[2]
gb|JX446542.1	Uncultured *Brachyspira sp*. clone	Patient_35Ib	partial sequence	Westerman et. al[2]
gb|JX446540.1	*Brachyspira aalborgi* clone	Patient_34	partial sequence	Westerman et. al[2]

Supplementary table 2. 16S sequences included as references - page 2

Accession number	Species	Name	16S type	Reference
gb|JX446539.1	*Brachyspira aalborgi* clone	Patient_33	partial sequence	Westerman et. al[2]
gb|JX446538.1	*Brachyspira aalborgi* clone	Patient_32	partial sequence	Westerman et. al[2]
gb|JX446537.1	*Brachyspira pilosicoli* clone	Patient_31	partial sequence	Westerman et. al[2]
gb|JX446536.1	*Brachyspira aalborgi* clone	Patient_30II	partial sequence	Westerman et. al[2]
gb|JX446535.1	*Brachyspira aalborgi* clone	Patient_30I	partial sequence	Westerman et. al[2]
gb|JX446534.1	*Brachyspira pilosicoli* clone	Patient_29	partial sequence	Westerman et. al[2]
gb|JX446533.1	*Brachyspira aalborgi* clone	Patient_28	partial sequence	Westerman et. al[2]
gb|JX446532.1	Uncultured *Brachyspira sp*. clone	Patient_27	partial sequence	Westerman et. al[2]
gb|JX446531.1	Uncultured *Brachyspira sp*. clone	Patient_26b	partial sequence	Westerman et. al[2]
gb|JX446529.1	*Brachyspira aalborgi* clone	Patient_25	partial sequence	Westerman et. al[2]
gb|JX446528.1	*Brachyspira aalborgi* clone	Patient_24	partial sequence	Westerman et. al[2]
gb|JX446527.1	*Brachyspira pilosicoli* clone	Patient_23	partial sequence	Westerman et. al[2]
gb|JX446526.1	Uncultured *Brachyspira sp*. clone	Patient_22	partial sequence	Westerman et. al[2]
gb|JX446525.1	*Brachyspira aalborgi* clone	Patient_21	partial sequence	Westerman et. al[2]
gb|JX446524.1	*Brachyspira pilosicoli* clone	Patient_20	partial sequence	Westerman et. al[2]
gb|JX446523.1	Uncultured *Brachyspira sp*. clone	Patient_19b	partial sequence	Westerman et. al[2]
gb|JX446521.1	*Brachyspira aalborgi* clone	Patient_18	partial sequence	Westerman et. al[2]
gb|JX446520.1	Uncultured *Brachyspira sp*. clone	Patient_17b	partial sequence	Westerman et. al[2]
gb|JX446518.1	Uncultured *Brachyspira sp*. clone	Patient_16	partial sequence	Westerman et. al[2]
gb|JX446517.1	Uncultured *Brachyspira sp*. clone	Patient_15b	partial sequence	Westerman et. al[2]
gb|JX446515.1	Uncultured *Brachyspira sp*. clone	Patient_14	partial sequence	Westerman et. al[2]
gb|JX446514.1	*Brachyspira aalborgi* clone	Patient_13	partial sequence	Westerman et. al[2]
gb|JX446513.1	*Brachyspira aalborgi* clone	Patient_12	partial sequence	Westerman et. al[2]
gb|JX446512.1	*Brachyspira aalborgi* clone	Patient_11	partial sequence	Westerman et. al[2]
gb|JX446511.1	*Brachyspira aalborgi* clone	Patient_10	partial sequence	Westerman et. al[2]
gb|JX446510.1	*Brachyspira aalborgi* clone	Patient_9II	partial sequence	Westerman et. al[2]
gb|JX446509.1	*Brachyspira aalborgi* clone	Patient_9I	partial sequence	Westerman et. al[2]
gb|JX446508.1	*Brachyspira aalborgi* clone	Patient_8	partial sequence	Westerman et. al[2]
gb|JX446507.1	*Brachyspira aalborgi* clone	Patient_7III	partial sequence	Westerman et. al[2]
gb|JX446506.1	*Brachyspira aalborgi* clone	Patient_7II	partial sequence	Westerman et. al[2]
gb|JX446505.1	Uncultured *Brachyspira sp*. clone	Patient_7Ib	partial sequence	Westerman et. al[2]
gb|JX446503.1	*Brachyspira pilosicoli* clone	Patient_6III	partial sequence	Westerman et. al[2]
gb|JX446502.1	*Brachyspira pilosicoli* clone	Patient_6I	partial sequence	Westerman et. al[2]
gb|JX446501.1	*Brachyspira aalborgi* clone	Patient_5	partial sequence	Westerman et. al[2]
gb|JX446500.1	*Brachyspira aalborgi* clone	Patient_4	partial sequence	Westerman et. al[2]
gb|JX446499.1	*Brachyspira pilosicoli* clone	Patient_3	partial sequence	Westerman et. al[2]
gb|JX446498.1	*Brachyspira aalborgi* clone	Patient_2	partial sequence	Westerman et. al[2]
gb|JX446497.1	*Brachyspira aalborgi* clone	Patient_1	partial sequence	Westerman et. al[2]
gb|JX463024.1	Uncultured *Brachyspira sp*. clone	Apndx_occ_141	partial sequence	Westerman et. al[2]
gb|JX463023.1	Uncultured *Brachyspira sp*. clone	Apndx_occ_131	partial sequence	Westerman et. al[2]
gb|JX463022.1	Uncultured *Brachyspira sp*. clone	Ps_apndx_acc_113	partial sequence	Westerman et. al[2]
gb|JX463021.1	Uncultured *Brachyspira sp*. clone	Ps_apndx_acc_111	partial sequence	Westerman et. al[2]
gb|JX463020.1	Uncultured *Brachyspira sp*. clone	Apndx_Acc_57	partial sequence	Westerman et. al[2]
gb|AF200693.1	*Brachyspira aalborgi* strain W1	W1	partial sequence	Pettersson et. al[3]
gb|AF228822.1	Uncultured bacterium	adhucomu_Tvb72	partial sequence	Pettersson et. al[3]
gb|AF228821.1	Uncultured bacterium	adhucomu_Tvb40	partial sequence	Pettersson et. al[3]
gb|AF228820.1	Uncultured bacterium	adhucomu_Tvb21	partial sequence	Pettersson et. al[3]
gb|AF228819.1	Uncultured bacterium	adhucomu_Tvb03	partial sequence	Pettersson et. al[3]
gb|AF228818.1	Uncultured bacterium	adhucomu_Tva35	partial sequence	Pettersson et. al[3]

Supplementary table 2. 16S sequences included as references - page 3

Accession number	Species	Name	16S type	Reference
gb|AF228817.1	Uncultured bacterium	adhucomu_Tva17	partial sequence	Pettersson et. al[3]
gb|AF228816.1	Uncultured bacterium	adhucomu_Hcc39	partial sequence	Pettersson et. al[3]
gb|AF228815.1	Uncultured bacterium	adhucomu_Hcc36	partial sequence	Pettersson et. al[3]
gb|AF228814.1	Uncultured bacterium	adhucomu_Hcc35	partial sequence	Pettersson et. al[3]
gb|AF228813.1	Uncultured bacterium	adhucomu_Hcc33	partial sequence	Pettersson et. al[3]
gb|AF228812.1	Uncultured bacterium	adhucomu_Hcc07	partial sequence	Pettersson et. al[3]
gb|AF228811.1	Uncultured bacterium	adhucomu_Hca76	partial sequence	Pettersson et. al[3]
gb|AF228810.1	Uncultured bacterium	adhucomu_Hca61	partial sequence	Pettersson et. al[3]
gb|AF228809.1	Uncultured bacterium	adhucomu_Hca41	partial sequence	Pettersson et. al[3]
gb|AF228808.1	Uncultured bacterium	adhucomu_Hca25	partial sequence	Pettersson et. al[3]
gb|AF228807.1	Uncultured bacterium	adhucomu_Hca20	partial sequence	Pettersson et. al[3]
gb|AF228806.1	Uncultured bacterium	adhucomu_Hca09	partial sequence	Pettersson et. al[3]
CAYT010000179	*Brachyspira sp.*	CAG700	partial sequence	SILVA database[4]
HE793032	*Brachyspira pilosicoli*	WesB	complete sequence	SILVA database[4]
CP002025	*Brachyspira pilosicoli*	95/1000	complete sequence	SILVA database[4]
CP002873	*Brachyspira pilosicoli*	P43/6/78	partial sequence	SILVA database[4]
CP003490	*Brachyspira pilosicoli*	B2904	partial sequence	SILVA database[4]

[1] Mikosza AS et. al: **Analysis of genetic variation in Brachyspira aalborgi and related spirochaetes determined by partial sequencing of the 16S rRNA and NADH oxidase genes.** *J Med Microbiol* 2004, **53:**333-339.

[2] Westerman LJ et. al: **Development of a real-time PCR for identification of brachyspira species in human colonic biopsies.** *PLoS One* 2012, **7:**e52281.

[3] Pettersson B et. al: **Phylogenetic evidence for novel and genetically different intestinal spirochetes resembling Brachyspira aalborgi in the mucosa of the human colon as revealed by 16S rDNA analysis.** *Syst Appl Microbiol* 2000, **23:**355-363.

[4] SILVA database, https://www.arb-silva.de/

Supplementary table 3. NADH oxidase (*nox*) sequences included as references

Accession number	Species	Strain/isolate	Description
AF060816.1	*Brachyspira aalborgi*	ATCC 43994	NADH oxidase gene, partial cds
KF386072.1	*Brachyspira alvinipulli*	AIS154	NADH oxidase (nox) gene, partial cds
KF386076.1	*Brachyspira alvinipulli*	AIS163	NADH oxidase (nox) gene, partial cds
KF386053.1	*Brachyspira alvinipulli*	AIS47	NADH oxidase (nox) gene, partial cds
KF386057.1	*Brachyspira alvinipulli*	AIS59	NADH oxidase (nox) gene, partial cds
KF386058.1	*Brachyspira alvinipulli*	AIS60	NADH oxidase (nox) gene, partial cds
KF386067.1	*Brachyspira alvinipulli*	AIS80	NADH oxidase (nox) gene, partial cds
JF430770.1	*Brachyspira alvinipulli*	AN3382/2/03	NADH oxidase (nox) gene, partial cds
JF430742.1	*Brachyspira alvinipulli*	AN3649/1/02	NADH oxidase (nox) gene, partial cds
JF430746.1	*Brachyspira alvinipulli*	AN3649/3b/02	NADH oxidase (nox) gene, partial cds
JF430743.1	*Brachyspira alvinipulli*	AN3933/1/02	NADH oxidase (nox) gene, partial cds
JF430747.1	*Brachyspira alvinipulli*	AN3940/1/02	NADH oxidase (nox) gene, partial cds
JF430771.1	*Brachyspira alvinipulli*	AN4578/01	NADH oxidase (nox) gene, partial cds
JF430744.1	*Brachyspira alvinipulli*	AN6041:1/1/00	NADH oxidase (nox) gene, partial cds
JF430745.1	*Brachyspira alvinipulli*	AN6050:2/1/00	NADH oxidase (nox) gene, partial cds
KR809396.1	*Brachyspira alvinipulli*	Ant10:1/1/09	NADH oxidase (nox) gene, partial cds
KR809403.1	*Brachyspira alvinipulli*	Ant226/3/12	NADH oxidase (nox) gene, partial cds
KR809404.1	*Brachyspira alvinipulli*	Ant230/3/12	NADH oxidase (nox) gene, partial cds
AF060814.1	*Brachyspira alvinipulli*	C1	NADH oxidase gene, partial cds
EU819071.1	*Brachyspira canis*	Dog A2	NADH oxidase (nox) gene, partial cds
KT381866.1	*Brachyspira canis*	RSG01	NADH oxidase gene, partial cds
KT381867.1	*Brachyspira canis*	RSG02	NADH oxidase gene, partial cds
KT381868.1	*Brachyspira canis*	RSG03	NADH oxidase gene, partial cds
KT381869.1	*Brachyspira canis*	RSG05	NADH oxidase gene, partial cds
EF436590.1	*Brachyspira canis*	SRBP205IR	NADH oxidase (nox) gene, partial cds
EU819072.1	*Brachyspira corvi*	AN1084/1/04	NADH oxidase (nox) gene, partial cds
EU819073.1	*Brachyspira corvi*	AN1865/1/04	NADH oxidase (nox) gene, partial cds
EU819074.1	*Brachyspira corvi*	AN1876/2/04	NADH oxidase (nox) gene, partial cds
EU819075.1	*Brachyspira corvi*	AN968/2/04	NADH oxidase (nox) gene, partial cds
KU215621.1	*Brachyspira hyodysenteriae*	49	NADH oxidase (nox) gene, partial cds
JX428806.1	*Brachyspira hyodysenteriae*	3140	NADH oxidase (nox) gene, partial cds
DQ487115.1	*Brachyspira hyodysenteriae*	AN 1409:2/01	NADH oxidase (nox) gene, partial cds
DQ487117.1	*Brachyspira hyodysenteriae*	AN 174/92	NADH oxidase (nox) gene, partial cds
DQ487118.1	*Brachyspira hyodysenteriae*	AN 2420/97	NADH oxidase (nox) gene, partial cds
DQ487116.1	*Brachyspira hyodysenteriae*	AN 383:2/00	NADH oxidase (nox) gene, partial cds
KC984311.1	*Brachyspira hyodysenteriae*	ATCC 27164	NADH oxidase (nox) gene, partial cds
KF680564.1	*Brachyspira hyodysenteriae*	B16	nox gene, partial sequence UNVERIFIED
AF060801.1	*Brachyspira hyodysenteriae*	B169	NADH oxidase gene, partial cds
KF680565.1	*Brachyspira hyodysenteriae*	B24	nox gene, partial sequence UNVERIFIED
KF680573.1	*Brachyspira hyodysenteriae*	B51	nox gene, partial sequence UNVERIFIED
KF680574.1	*Brachyspira hyodysenteriae*	B53	nox gene, partial sequence UNVERIFIED
KF680575.1	*Brachyspira hyodysenteriae*	B60	nox gene, partial sequence UNVERIFIED
AF060800.1	*Brachyspira hyodysenteriae*	B78	NADH oxidase gene, partial cds
KF680580.1	*Brachyspira hyodysenteriae*	B81	nox gene, partial sequence UNVERIFIED
AF060802.1	*Brachyspira hyodysenteriae*	R1	NADH oxidase gene, partial cds
HM462461.1	*Brachyspira hyodysenteriae*		NADH oxidase (nox) gene, partial cds
AF060810.1	*Brachyspira intermedia*	2818.5	NADH oxidase gene, partial cds
AF060812.1	*Brachyspira intermedia*	4482	NADH oxidase gene, partial cds
AH015259.2	*Brachyspira intermedia*	1296/02	NADH oxidase (nox) gene, partial cds
JF430736.1	*Brachyspira intermedia*	AN3649/7b/02	NADH oxidase (nox) gene, partial cds
JF430737.1	*Brachyspira intermedia*	AN3651/1b/02	NADH oxidase (nox) gene, partial cds
JF430738.1	*Brachyspira intermedia*	AN3651/2b/02	NADH oxidase (nox) gene, partial cds
EF517542.1	*Brachyspira intermedia*	AN519/97	nox (nox) gene, partial cds
EF517543.1	*Brachyspira intermedia*	AN983/90	nox gene, partial sequence
KR809402.1	*Brachyspira intermedia*	Ant226/1/12	NADH oxidase (nox) gene, partial cds
KF680562.1	*Brachyspira intermedia*	B113	nox gene, partial sequence UNVERIFIED
KF680566.1	*Brachyspira intermedia*	B25	nox gene, partial sequence UNVERIFIED
KF680576.1	*Brachyspira intermedia*	B67	nox gene, partial sequence UNVERIFIED
KF680577.1	*Brachyspira intermedia*	B69	nox gene, partial sequence UNVERIFIED
MF116417.1	*Brachyspira intermedia*	JB1	NADH oxidase (nox) gene, partial cds
AF060811.1	*Brachyspira intermedia*	PWS/A	NADH oxidase gene, partial cds
CP002874.1	*Brachyspira intermedia*	PWS/A	complete genome
KC984309.1	*Brachyspira intermedia*	PWS/A, strain ATCC 51140	NADH oxidase (nox) gene, partial cds
KY216078.1	*Brachyspira intermedia*	T1.6	NADH oxidase (nox) gene, partial cds
KY216086.1	*Brachyspira intermedia*	T9.4	NADH oxidase (nox) gene, partial cds
KY216087.1	*Brachyspira intermedia*	T9.5	NADH oxidase (nox) gene, partial cds
JX428829.1	*Brachyspira intermedia*	X26	NADH oxidase (nox) gene, partial cds
JX428825.1	*Brachyspira intermedia*	X31	NADH oxidase (nox) gene, partial cds
JX428826.1	*Brachyspira intermedia*	X45	NADH oxidase (nox) gene, partial cds
JX428828.1	*Brachyspira intermedia*	X53	NADH oxidase (nox) gene, partial cds

Supplementary table 3. NADH oxidase (*nox*) sequences included as references - page 2

Accession number	Species	Strain/isolate	Description
JX428827.1	*Brachyspira intermedia*	XCL2	NADH oxidase (nox) gene, partial cds
DQ458796.1	*Brachyspira intermedia*		NADH oxidase (nox) gene, partial cds
JF430769.1	*Brachyspira pulli*	AN304/04	NADH oxidase (nox) gene, partial cds
JF430741.1	*Brachyspira pulli*	AN3651/3b/02	NADH oxidase (nox) gene, partial cds
JF430739.1	*Brachyspira pulli*	AN6045/1/00	NADH oxidase (nox) gene, partial cds
JF430740.1	*Brachyspira pulli*	AN6052:2/1/00	NADH oxidase (nox) gene, partial cds
KR809397.1	*Brachyspira pulli*	Ant10:1/2/09	NADH oxidase (nox) gene, partial cds
KR809401.1	*Brachyspira pulli*	Ant25:2/1/09	NADH oxidase (nox) gene, partial cds
JX428824.1	*Brachyspira pulli*	H48	NADH oxidase (nox) gene, partial cds
MF116419.1	*Brachyspira pulli*	JB2	NADH oxidase (nox) gene, partial cds
KY216084.1	*Brachyspira pulli*	T7.5	NADH oxidase (nox) gene, partial cds
KY216085.1	*Brachyspira pulli*	T8.5	NADH oxidase (nox) gene, partial cds
KY216091.1	*Brachyspira pulli*	W1.5	NADH oxidase (nox) gene, partial cds
KY216102.1	*Brachyspira pulli*	W4.2	NADH oxidase (nox) gene, partial cds
KY216103.1	*Brachyspira pulli*	W4.3	NADH oxidase (nox) gene, partial cds
HM462456.1	*Brachyspira sp.*	9757/1	NADH oxidase (nox) gene, partial cds
HM462458.1	*Brachyspira sp.*	10363/4	NADH oxidase (nox) gene, partial cds
HM462459.1	*Brachyspira sp.*	21330/2	NADH oxidase (nox) gene, partial cds
HM462462.1	*Brachyspira sp.*	24916/1	NADH oxidase (nox) gene, partial cds
KC561393.1	*Brachyspira sp.*	A22	NADH oxidase gene, partial cds
KC561379.1	*Brachyspira sp.*	A26	NADH oxidase gene, partial cds
KC561381.1	*Brachyspira sp.*	A27	NADH oxidase gene, partial cds
KC561391.1	*Brachyspira sp.*	A50	NADH oxidase gene, partial cds
KC561431.1	*Brachyspira sp.*	A55	NADH oxidase gene, partial cds
KC561392.1	*Brachyspira sp.*	A58	NADH oxidase gene, partial cds
KC561434.1	*Brachyspira sp.*	A59	NADH oxidase gene, partial cds
KC561427.1	*Brachyspira sp.*	A60	NADH oxidase gene, partial cds
KC561433.1	*Brachyspira sp.*	A61	NADH oxidase gene, partial cds
KC561398.1	*Brachyspira sp.*	A62	NADH oxidase gene, partial cds
KC561396.1	*Brachyspira sp.*	A63	NADH oxidase gene, partial cds
DQ487125.1	*Brachyspira sp.*	AN 3930:2/02	NADH oxidase (nox) gene, partial cds
JF430768.1	*Brachyspira sp.*	AN3442/1/02	NADH oxidase (nox) gene, partial cds
JF430758.1	*Brachyspira sp.*	AN3617:2/1/02	NADH oxidase (nox) gene, partial cds
JF430735.1	*Brachyspira sp.*	AN3649/2b/02	NADH oxidase (nox) gene, partial cds
JF430734.1	*Brachyspira sp.*	AN6043:2/1/00	NADH oxidase (nox) gene, partial cds
KR809398.1	*Brachyspira sp.*	Ant10:2a/1/09	NADH oxidase (nox) gene, partial cds
KR809395.1	*Brachyspira sp.*	Ant112/1/02	NADH oxidase (nox) gene, partial cds
KR809399.1	*Brachyspira sp.*	Ant16/1/09	NADH oxidase (nox) gene, partial cds
KR809405.1	*Brachyspira sp.*	Ant235/1/12	NADH oxidase (nox) gene, partial cds
KR809406.1	*Brachyspira sp.*	Ant357/1/12	NADH oxidase (nox) gene, partial cds
KC561424.1	*Brachyspira sp.*	B10	NADH oxidase gene, partial cds
KC561405.1	*Brachyspira sp.*	B31	NADH oxidase gene, partial cds
KC561428.1	*Brachyspira sp.*	B32	NADH oxidase gene, partial cds
KC561450.1	*Brachyspira sp.*	B34	NADH oxidase gene, partial cds
KC561387.1	*Brachyspira sp.*	B4	NADH oxidase gene, partial cds
KC561426.1	*Brachyspira sp.*	B56a	NADH oxidase gene, partial cds
KC561429.1	*Brachyspira sp.*	B56c	NADH oxidase gene, partial cds
KC561425.1	*Brachyspira sp.*	B57	NADH oxidase gene, partial cds
KC561401.1	*Brachyspira sp.*	B58	NADH oxidase gene, partial cds
KC561413.1	*Brachyspira sp.*	B60	NADH oxidase gene, partial cds
KC561411.1	*Brachyspira sp.*	B64	NADH oxidase gene, partial cds
KC561388.1	*Brachyspira sp.*	B66	NADH oxidase gene, partial cds
KC561395.1	*Brachyspira sp.*	B7	NADH oxidase gene, partial cds
KC561412.1	*Brachyspira sp.*	B70	NADH oxidase gene, partial cds
KC561422.1	*Brachyspira sp.*	B71	NADH oxidase gene, partial cds
KC561430.1	*Brachyspira sp.*	B78	NADH oxidase gene, partial cds
KC561373.1	*Brachyspira sp.*	C32	NADH oxidase gene, partial cds
KC561402.1	*Brachyspira sp.*	C35	NADH oxidase gene, partial cds
KC561435.1	*Brachyspira sp.*	C44	NADH oxidase gene, partial cds
KC561415.1	*Brachyspira sp.*	C47	NADH oxidase gene, partial cds
KC561407.1	*Brachyspira sp.*	C52	NADH oxidase gene, partial cds
KC561403.1	*Brachyspira sp.*	C61	NADH oxidase gene, partial cds
KC561437.1	*Brachyspira sp.*	C68	NADH oxidase gene, partial cds
KC561382.1	*Brachyspira sp.*	C72	NADH oxidase gene, partial cds
KC561442.1	*Brachyspira sp.*	C75	NADH oxidase gene, partial cds
KC878394.1	*Brachyspira sp.*	CH-003	NADH oxidase (nox) gene, partial cds
KC878395.1	*Brachyspira sp.*	CH-013	NADH oxidase (nox) gene, partial cds
KC878396.1	*Brachyspira sp.*	CH-014	NADH oxidase (nox) gene, partial cds
KC878397.1	*Brachyspira sp.*	CH-108	NADH oxidase (nox) gene, partial cds
KC878398.1	*Brachyspira sp.*	CH-110	NADH oxidase (nox) gene, partial cds

Supplementary table 3. NADH oxidase (*nox*) sequences included as references - page 3

Accession number	Species	Strain/isolate	Description
KC561383.1	*Brachyspira sp.*	D03	NADH oxidase gene, partial cds
KC561443.1	*Brachyspira sp.*	D08	NADH oxidase gene, partial cds
KC561389.1	*Brachyspira sp.*	D11	NADH oxidase gene, partial cds
KC561420.1	*Brachyspira sp.*	D61	NADH oxidase gene, partial cds
KC561408.1	*Brachyspira sp.*	D71	NADH oxidase gene, partial cds
KC561432.1	*Brachyspira sp.*	D75	NADH oxidase gene, partial cds
KC561451.1	*Brachyspira sp.*	D85	NADH oxidase gene, partial cds
KC561449.1	*Brachyspira sp.*	E35	NADH oxidase gene, partial cds
KC561421.1	*Brachyspira sp.*	E62	NADH oxidase gene, partial cds
KC561440.1	*Brachyspira sp.*	E75	NADH oxidase gene, partial cds
KC561445.1	*Brachyspira sp.*	F01	NADH oxidase gene, partial cds
KC561438.1	*Brachyspira sp.*	F07	NADH oxidase gene, partial cds
KC561439.1	*Brachyspira sp.*	F08	NADH oxidase gene, partial cds
KC561385.1	*Brachyspira sp.*	F21	NADH oxidase gene, partial cds
KC561423.1	*Brachyspira sp.*	F31	NADH oxidase gene, partial cds
KC561446.1	*Brachyspira sp.*	F32	NADH oxidase gene, partial cds
KC561447.1	*Brachyspira sp.*	F33	NADH oxidase gene, partial cds
KC561394.1	*Brachyspira sp.*	F52	NADH oxidase gene, partial cds
KC561400.1	*Brachyspira sp.*	F56	NADH oxidase gene, partial cds
KC561414.1	*Brachyspira sp.*	F62	NADH oxidase gene, partial cds
KC561416.1	*Brachyspira sp.*	F65	NADH oxidase gene, partial cds
KC561409.1	*Brachyspira sp.*	F66	NADH oxidase gene, partial cds
KC561406.1	*Brachyspira sp.*	F68	NADH oxidase gene, partial cds
KC561384.1	*Brachyspira sp.*	F73	NADH oxidase gene, partial cds
KC561444.1	*Brachyspira sp.*	F80	NADH oxidase gene, partial cds
KC561452.1	*Brachyspira sp.*	F83	NADH oxidase gene, partial cds
KC561441.1	*Brachyspira sp.*	F85	NADH oxidase gene, partial cds
KC561404.1	*Brachyspira sp.*	F87	NADH oxidase gene, partial cds
KC561378.1	*Brachyspira sp.*	G21	NADH oxidase gene, partial cds
KC561374.1	*Brachyspira sp.*	G26	NADH oxidase gene, partial cds
KC561419.1	*Brachyspira sp.*	G62	NADH oxidase gene, partial cds
KC561417.1	*Brachyspira sp.*	G63	NADH oxidase gene, partial cds
KC561399.1	*Brachyspira sp.*	G70	NADH oxidase gene, partial cds
KC561410.1	*Brachyspira sp.*	G81	NADH oxidase gene, partial cds
KC561453.1	*Brachyspira sp.*	GZZ	NADH oxidase gene, partial cds
JX428805.1	*Brachyspira sp.*	H29	NADH oxidase (nox) gene, partial cds
KC561436.1	*Brachyspira sp.*	H45	NADH oxidase gene, partial cds
KC561418.1	*Brachyspira sp.*	H58	NADH oxidase gene, partial cds
KC561377.1	*Brachyspira sp.*	H73	NADH oxidase gene, partial cds
KC561376.1	*Brachyspira sp.*	H87	NADH oxidase gene, partial cds
KC561390.1	*Brachyspira sp.*	I12	NADH oxidase gene, partial cds
KC561448.1	*Brachyspira sp.*	I14	NADH oxidase gene, partial cds
KC561380.1	*Brachyspira sp.*	J11	NADH oxidase gene, partial cds
KC561375.1	*Brachyspira sp.*	J13	NADH oxidase gene, partial cds
KC561397.1	*Brachyspira sp.*	K07	NADH oxidase gene, partial cds
KC878399.1	*Brachyspira sp.*	KL-049	NADH oxidase (nox) gene, partial cds
KC878400.1	*Brachyspira sp.*	KL-169	NADH oxidase (nox) gene, partial cds
KC878402.1	*Brachyspira sp.*	KL-181	NADH oxidase (nox) gene, partial cds
KC878403.1	*Brachyspira sp.*	KL-194	NADH oxidase (nox) gene, partial cds
KC878404.1	*Brachyspira sp.*	KL-207	NADH oxidase (nox) gene, partial cds
KC561386.1	*Brachyspira sp.*	L20	NADH oxidase gene, partial cds
JX232302.1	*Brachyspira sp.*	NSH-1	NADH oxidase (nox) gene, partial cds
JX232322.1	*Brachyspira sp.*	NSH-28	NADH oxidase (nox) gene, partial cds
JX232304.1	*Brachyspira sp.*	NSH-3	NADH oxidase (nox) gene, partial cds
JX232324.1	*Brachyspira sp.*	NSH-30	NADH oxidase (nox) gene, partial cds
JX232327.1	*Brachyspira sp.*	NSH-33	NADH oxidase (nox) gene, partial cds
JX232328.1	*Brachyspira sp.*	NSH-34	NADH oxidase (nox) gene, partial cds
JX232329.1	*Brachyspira sp.*	NSH-35	NADH oxidase (nox) gene, partial cds
JX232331.1	*Brachyspira sp.*	NSH-37	NADH oxidase (nox) gene, partial cds
JX232332.1	*Brachyspira sp.*	NSH-38	NADH oxidase (nox) gene, partial cds
JX232333.1	*Brachyspira sp.*	NSH-39	NADH oxidase (nox) gene, partial cds
JX232309.1	*Brachyspira sp.*	NSH-8	NADH oxidase (nox) gene, partial cds
KC878405.1	*Brachyspira sp.*	SHI-031	NADH oxidase (nox) gene, partial cds
KC878406.1	*Brachyspira sp.*	SHI-067	NADH oxidase (nox) gene, partial cds
KC878407.1	*Brachyspira sp.*	SHI-068	NADH oxidase (nox) gene, partial cds
KC878408.1	*Brachyspira sp.*	SHI-129	NADH oxidase (nox) gene, partial cds
KY798219.1	*Brachyspira suanatina*	755-3x 17	NADH oxidase (nox) gene, partial cds
DQ487124.1	*Brachyspira suanatina*	AN 1418:2/01	NADH oxidase (nox) gene, partial cds
DQ487120.1	*Brachyspira suanatina*	AN 1681:1/04	NADH oxidase (nox) gene, partial cds
DQ487121.1	*Brachyspira suanatina*	AN 2384/04	NADH oxidase (nox) gene, partial cds

Supplementary table 3. NADH oxidase (*nox*) sequences included as references - page 4

Accession number	Species	Strain/isolate	Description
DQ487123.1	*Brachyspira suanatina*	AN 3949:2/02	NADH oxidase (nox) gene, partial cds
DQ487119.1	*Brachyspira suanatina*	AN 4859/03	NADH oxidase (nox) gene, partial cds
DQ487122.1	*Brachyspira suanatina*	Dk12570-2	NADH oxidase (nox) gene, partial cds
HM462463.1	*Brachyspira suanatina*		NADH oxidase (nox) gene, partial cds

Supplementary table 4. Sequencing statistics

	Isolate	Number of contigs	Total length	Min length	max length	mean length	median length	N50	Coverage
	PC2022III	15	2609344	971	671466	173956.267	91392	355956	374
	PC2777IV	30	2655229	513	521706	88507.6333	3784	212275	393
	PC3053II	34	2667320	507	596308	78450.5882	4755	221305	353
	PC3517II	35	2645532	513	422249	75586.6286	6467	279397	361
	PC3714II	24	2743156	557	742608	114298.167	45756	288252	386
	PC390II	32	2660153	507	586895	83129.7813	16877	282401	351
	PC3939II	24	2589393	627	589876	107891.375	25715	317138	433
	PC3997IV	33	2649511	537	938493	80288.2121	5000	576737	620
Clinical Isolates	PC4226IV	40	2715122	502	457548	67878.05	23603	197907	159
	PC4580III	10	2591031	660	1066433	259103.1	177803	571723	482
	PC4597II	28	2670785	551	980309	95385.1786	22586	311232	339
	PC5099IV	21	2714460	502	674763	129260	28013	390143	321
	PC5538III-hc	113	2760914	506	129964	24218.5	12067	56982	
	PC5538III-lc	32	2746864	510	277228	85839.5	61847	195041	
	PC5587-p	33	2665010	551	976313	80757.8788	3345	276929	320
	PC5587-u	32	2662929	551	976207	83216.5313	6064	202399	476
Type strain	*513A*^{T}	8	2504147	554	2486925	313018.375	1436	2486925	433
Ref. strain	W1	15	2666344	520	827193	177756.267	24070	670021	517

Supplementary table 5. Human Microbiome Project (HMP) samples analysed

SRA accession	Subject	Body Site	WGS	Amplified region	Perc. Brachyspira	Brachyspira in assembly
SRS055563	132902142	stool		V1-V3	0%	
SRS058416	147406386	stool		V3-V5	0%	
SRS021601	158013734	stool		V1-V3	0%	
SRS012191	158013734	stool		V3-V5	0%	
SRS022987	158114885	stool		V1-V3	0%	
SRS021910	158155345	stool		V1-V3	0%	
SRS021541	158216035	stool		V3-V5	0%	
SRS022924	158236265	stool		V1-V3	0%	
SRS021970	158256496	stool		V3-V5	0%	
SRS023047	158276726	stool		V1-V3	0%	
SRS022093	158337416	stool		V3-V5	0%	
SRS017935	158357646	stool		V3-V5	0%	
SRS020119	158398106	stool		V1-V3	0%	
SRS020176	158418336	stool		V3-V5	0%	
SRS023422	158438567	stool		V3-V5	0%	
SRS022480	158458797	stool		V1-V3	0%	
SRS021484	158479027	stool	X	V3-V5	0%	
SRS022609	158499257	stool	X	V3-V5	0%	
SRS023734	158721788	stool		V3-V5	0%	
SRS022414	158742018	stool		V3-V5	0%	
SRS011271	158802708	stool	X	V1-V3	0%	
SRS023545	158802708	stool		V3-V5	0%	
SRS011157	158822939	stool		V3-V5	0%	
SRS023851	158883629	stool		V1-V3	0%	
SRS011300	158924089	stool		V1-V3	0%	
SRS023176	158924089	stool	X	V3-V5	0%	
SRS024265	158944319	stool	X	V3-V5	0%	
SRS023488	158964549	stool		V1-V3	0%	
SRS011410	158964549	stool		V3-V5	0%	
SRS011413	159005010	stool		V3-V5	0%	
SRS023605	159005010	stool		V3-V5	0%	
SRS011415	159085930	stool		V3-V5	0%	
SRS042290	159085930	stool		V3-V5	0%	
SRS011452	159146620	stool	X	V1-V3	0%	
SRS048083	159146620	stool		V3-V5	0%	
SRS011529	159166850	stool	X	V1-V3	0%	
SRS057122	159166850	stool		V1-V3	0%	
SRS013490	159207311	stool		V3-V5	0%	
SRS024151	159207311	stool		V3-V5	0%	
SRS013543	159227541	stool		V3-V5	0%	
SRS050599	159227541	stool		V3-V5	0%	
SRS011405	159247771	stool	X	V3-V5	0%	
SRS024028	159247771	stool		V3-V5	0%	
SRS013687	159268001	stool	X	V3-V5	0%	
SRS023914	159268001	stool	X	V3-V5	0%	
SRS013638	159288231	stool		V3-V5	0%	
SRS056656	159288231	stool		V3-V5	0%	
SRS016841	159308461	stool		V3-V5	0%	
SRS024208	159308461	stool		V3-V5	0%	
SRS013386	159328691	stool		V3-V5	0%	
SRS013177	159369152	stool		V3-V5	0%	
SRS023971	159369152	stool	X	V3-V5	0%	
SRS013762	159389382	stool		V1-V3	0%	

Supplementary table 5. Human Microbiome Project (HMP) samples analysed - page 2

SRA accession	Subject	Body Site	WGS	Amplified region	Perc. Brachyspira	Brachyspira in assembly
SRS024511	159389382	stool		V3-V5	0%	
SRS016437	159429842	stool		V3-V5	0%	
SRS016916	159450072	stool		V3-V5	0%	
SRS049911	159450072	stool		V3-V5	0%	
SRS011621	159470302	stool		V1-V3	0%	
SRS016990	159490532	stool		V3-V5	0%	
SRS057901	159490532	stool		V3-V5	0%	
SRS017266	159510762	stool		V3-V5	0%	
SRS024094	159510762	stool		V3-V5	0%	
SRS063797	159510762	stool		V3-V5	0%	
SRS017103	159551223	stool	X	V3-V5	0%	
SRS024331	159551223	stool	X	V3-V5	0%	
SRS013801	159571453	stool		V3-V5	0%	
SRS024454	159571453	stool		V3-V5	0%	
SRS013216	159591683	stool		V3-V5	0%	
SRS024568	159591683	stool		V3-V5	0%	
SRS016954	159611913	stool	X	V3-V5	0%	
SRS024625	159611913	stool	X	V3-V5	0%	
SRS017307	159632143	stool	X	V3-V5	0%	
SRS054488	159632143	stool		V3-V5	0%	
SRS016400	159672603	stool		V3-V5	0%	
SRS053956	159672603	stool		V3-V5	0%	
SRS011653	159713063	stool		V3-V5	0%	
SRS024388	159713063	stool	X	V3-V5	0%	
SRS011586	159733294	stool	X	V1-V3	0%	
SRS016517	159753524	stool	X	V3-V5	0%	
SRS056505	159753524	stool		V3-V5	0%	
SRS017191	159814214	stool	X	V3-V5	0%	
SRS043701	159814214	stool	X	V3-V5	0%	
SRS016585	159915365	stool	X	V3-V5	0%	
SRS055934	159915365	stool		V3-V5	0%	
SRS018068	160016515	stool		V3-V5	0%	
SRS016630	160036745	stool		V3-V5	0%	
SRS018076	160056975	stool		V3-V5	0%	
SRS045627	160056975	stool		V3-V5	0%	
SRS018190	160097436	stool		V3-V5	0%	
SRS042843	160097436	stool		V3-V5	0%	
SRS018133	160158126	stool	X	V3-V5	0%	
SRS058770	160158126	stool	X	V3-V5	0%	
SRS018370	160178356	stool		V3-V5	0%	
SRS055137	160178356	stool		V3-V5	0%	
SRS018427	160218816	stool	X	V3-V5	0%	
SRS042436	160218816	stool		V3-V5	0%	
SRS017997	160239046	stool		V3-V5	0%	
SRS018489	160259276	stool		V3-V5	0%	
SRS017455	160319967	stool		V3-V5	0%	
SRS045493	160319967	stool		V3-V5	0%	
SRS018313	160380657	stool	X	V3-V5	0%	
SRS048299	160380657	stool		V3-V5	0%	
SRS020233	160400887	stool	X	V3-V5	0%	
SRS051609	160400887	stool		V3-V5	0%	
SRS016753	160421117	stool	X	V3-V5	0%	
SRS045350	160421117	stool		V3-V5	0%	

Supplementary table 5. Human Microbiome Project (HMP) samples analysed - page 3

SRA accession	Subject	Body Site	WGS	Amplified region	Perc. Brachyspira	Brachyspira in assembly
SRS017878	160441347	stool		V3-V5	0%	
SRS017641	160461578	stool		V3-V5	0%	
SRS017394	160481808	stool		V3-V5	0%	
SRS017521	160502038	stool	X	V3-V5	0%	
SRS056255	160502038	stool		V3-V5	0%	
SRS017764	160542498	stool		V3-V5	0%	
SRS017701	160582958	stool	X	V3-V5	0%	
SRS047561	160582958	stool		V3-V5	0%	
SRS017821	160603188	stool	X	V3-V5	0%	
SRS052555	160603188	stool		V3-V5	0%	
SRS019990	160643649	stool		V3-V5	0%	
SRS049982	160643649	stool		V3-V5	0%	
SRS020290	160663879	stool		V3-V5	0%	
SRS020413	160684109	stool		V3-V5	0%	
SRS020350	160704339	stool		V3-V5	0%	
SRS050141	160704339	stool		V3-V5	0%	
SRS021109	160744799	stool		V1-V3	0%	
SRS020869	160765029	stool	X	V3-V5	0%	
SRS047967	160765029	stool		V3-V5	0%	
SRS020470	160825720	stool		V1-V3	0%	
SRS045910	160825720	stool		V3-V5	0%	
SRS020527	160845950	stool		V3-V5	0%	
SRS053301	160845950	stool		V3-V5	0%	
SRS021065	160866180	stool		V1-V3	0%	
SRS057258	160866180	stool		V3-V5	0%	
SRS020811	160886410	stool		V1-V3	0%	
SRS020584	160906640	stool		V1-V3	0%	
SRS020641	160947100	stool		V1-V3	0%	
SRS051086	160947100	stool		V3-V5	0%	
SRS021427	160967330	stool		V3-V5	0%	
SRS049949	160967330	stool		V3-V5	0%	
SRS020698	160987560	stool		V3-V5	0%	
SRS043769	160987560	stool		V3-V5	0%	
SRS042572	161007791	stool		V1-V3	0%	
SRS021175	161007791	stool		V3-V5	0%	
SRS021364	161028021	stool		V3-V5	0%	
SRS020926	161068481	stool		V3-V5	0%	
SRS021241	161230322	stool		V3-V5	0%	
SRS021853	161270782	stool		V1-V3	0%	
SRS056832	161270782	stool		V1-V3	0%	
SRS020983	161311242	stool		V3-V5	0%	
SRS046216	161311242	stool		V3-V5	0%	
SRS049766	161331472	stool		V1-V3	0%	
SRS021664	161331472	stool		V3-V5	0%	
SRS021304	161351702	stool		V3-V5	0%	
SRS053525	161351702	stool		V3-V5	0%	
SRS020755	161412393	stool		V3-V5	0%	
SRS044088	161412393	stool		V3-V5	0%	
SRS021790	161473083	stool		V3-V5	0%	
SRS051892	161473083	stool		V3-V5	0%	
SRS021724	161493313	stool		V3-V5	0%	
SRS022033	161554003	stool		V3-V5	0%	
SRS055727	161554003	stool		V3-V5	0%	

Supplementary table 5. Human Microbiome Project (HMP) samples analysed - page 4

SRA accession	Subject	Body Site	WGS	Amplified region	Perc. Brachyspira	Brachyspira in assembly
SRS054608	178713055	stool		V1-V3	0%	
SRS043299	184349034	stool		V3-V5	0%	
SRS023302	206906765	stool		V3-V5	0%	
SRS048722	208027353	stool		V3-V5	0%	
SRS023365	246515023	stool		V3-V5	0%	
SRS042365	246515023	stool		V3-V5	0%	
SRS063138	257905678	stool		V3-V5	0%	
SRS048838	275382046	stool		V1-V3	0%	
SRS046382	289996019	stool		V1-V3	0%	
SRS062847	289996019	stool		V1-V3	0%	
SRS063608	295137534	stool		V1-V3	0%	
SRS022798	295137534	stool		V3-V5	0%	
SRS023791	336497421	stool		V3-V5	0%	
SRS063918	336497421	stool		V3-V5	0%	
SRS054590	338793263	stool	X	V3-V5	0%	
SRS057267	355657046	stool		V3-V5	0%	
SRS024682	368533040	stool		V3-V5	0%	
SRS065628	368533040	stool		V3-V5	0%	
SRS046349	370027359	stool		V1-V3	0%	
SRS022735	370425937	stool		V3-V5	0%	
SRS042803	370425937	stool		V3-V5	0%	
SRS052196	375450439	stool		V1-V3	0%	
SRS063275	375450439	stool		V1-V3	0%	
SRS043804	404239096	stool		V3-V5	0%	
SRS064008	414519462	stool		V1-V3	0%	
SRS049157	414519462	stool		V3-V5	0%	
SRS022669	432193348	stool		V3-V5	0%	
SRS023106	441369442	stool		V3-V5	0%	
SRS064335	451588811	stool		V3-V5	0%	
SRS044415	465578759	stool		V3-V5	0%	
SRS022354	492786515	stool		V3-V5	0%	
SRS063968	492786515	stool		V3-V5	0%	
SRS053335	508703490	stool	X	V1-V3	0%	
SRS048008	514014184	stool		V3-V5	0%	
SRS046313	516889361	stool		V3-V5	0%	
SRS042415	517810313	stool		V3-V5	0%	
SRS022291	533247696	stool		V3-V5	0%	
SRS056620	550534656	stool		V1-V3	0%	
SRS045613	553359145	stool		V1-V3	0%	
SRS065665	561079058	stool		V1-V3	0%	
SRS047950	604812005	stool		V3-V5	0%	
SRS055697	604812005	stool		V3-V5	0%	
SRS055482	612472597	stool		V1-V3	0%	
SRS022159	638754422	stool		V3-V5	0%	
SRS057676	638754422	stool		V3-V5	0%	
SRS050733	643185023	stool		V3-V5	0%	
SRS063807	650853796	stool		V1-V3	0%	
SRS022858	650853796	stool		V3-V5	0%	
SRS065466	663835652	stool		V3-V5	0%	
SRS057447	668248235	stool		V1-V3	0%	
SRS054461	675950834	stool		V1-V3	0%	
SRS065725	682102541	stool		V1-V3	0%	
SRS052433	682449369	stool		V1-V3	0%	

Supplementary table 5. Human Microbiome Project (HMP) samples analysed - page 5

SRA accession	Subject	Body Site	WGS	Amplified region	Perc. Brachyspira	Brachyspira in assembly
SRS064647	686765762	stool		V1-V3	0%	
SRS047642	686765762	stool		V3-V5	0%	
SRS052697	706846339	stool	X	V1-V3	0%	
SRS045414	737052003	stool		V3-V5	0%	
SRS063324	739574095	stool		V1-V3	0%	
SRS065263	763395383	stool		V1-V3	0%	
SRS015815	763435843	stool		V3-V5	0%	
SRS045526	763456073	stool		V3-V5	0%	
SRS046341	763476303	stool		V3-V5	0%	
SRS054928	763516763	stool		V1-V3	0%	
SRS014369	763516763	stool		V3-V5	0%	
SRS014287	763536994	stool	X	V1-V3	0%	
SRS050422	763536994	stool	X	V1-V3	0%	
SRS062427	763536994	stool	X	V3-V5	0%	
SRS046639	763557224	stool		V3-V5	0%	
SRS014659	763597684	stool		V3-V5	0%	
SRS019582	763597684	stool	X	V3-V5	0%	
SRS014572	763638144	stool		V3-V5	0%	
SRS063898	763638144	stool		V3-V5	0%	
SRS014235	763678604	stool	X	V3-V5	0%	
SRS019685	763678604	stool	X	V3-V5	0%	
SRS014442	763698834	stool		V1-V3	0%	
SRS048819	763698834	stool		V3-V5	0%	
SRS063921	763719065	stool		V1-V3	0%	
SRS062610	763721271	stool		V3-V5	0%	
SRS015190	763759525	stool	X	V1-V3	0%	
SRS043411	763759525	stool	X	V1-V3	0%	
SRS052326	763820215	stool		V1-V3	0%	
SRS014345	763820215	stool		V3-V5	0%	
SRS014613	763840445	stool	X	V1-V3	0%	
SRS064276	763840445	stool	X	V3-V5	0%	
SRS014923	763860675	stool	X	V1-V3	0%	
SRS049428	763860675	stool		V1-V3	0%	
SRS014978	763880905	stool		V3-V5	0%	
SRS049620	763880905	stool		V3-V5	0%	
SRS015133	763901136	stool	X	V1-V3	0%	
SRS063985	763901136	stool	X	V1-V3	0%	
SRS014823	763921366	stool		V3-V5	0%	
SRS045877	763982056	stool		V1-V3	0%	
SRS015281	763982056	stool		V3-V5	0%	
SRS015854	764002286	stool	X	V1-V3	0%	
SRS014885	764002286	stool		V3-V5	0%	
SRS064557	764042746	stool	X	V1-V3	0%	
SRS015389	764042746	stool		V3-V5	0%	
SRS014999	764062976	stool		V3-V5	0%	
SRS015452	764083206	stool		V3-V5	0%	
SRS019089	764083206	stool		V6-V9	0%	
SRS015247	764143897	stool		V1-V3	0%	
SRS055665	764143897	stool		V1-V3	0%	
SRS015332	764184357	stool		V3-V5	0%	
SRS048870	764184357	stool	X	V3-V5	0%	
SRS049748	764224817	stool		V1-V3	0%	
SRS015782	764224817	stool	X	V3-V5	0%	

Supplementary table 5. Human Microbiome Project (HMP) samples analysed - page 6

SRA accession	Subject	Body Site	WGS	Amplified region	Perc. Brachyspira	Brachyspira in assembly
SRS015518	764245047	stool		V3-V5	0%	
SRS015578	764285508	stool	X	V1-V3	0%	
SRS015911	764305738	stool		V1-V3	0%	
SRS019013	764305738	stool		V3-V5	0%	
SRS056259	764325968	stool	X	V1-V3	0.036%	X
SRS015960	764325968	stool	X	V3-V5	0.033%	X
SRS015599	764346198	stool		V1-V3	0%	
SRS064233	764346198	stool		V3-V5	0%	
SRS016152	764366428	stool		V3-V5	0%	
SRS016018	764447348	stool	X	V1-V3	0%	
SRS042628	764447348	stool	X	V1-V3	0%	
SRS015724	764467579	stool		V1-V3	0%	
SRS051031	764487809	stool	X	V1-V3	0%	
SRS015663	764487809	stool	X	V3-V5	0%	
SRS016056	764508039	stool	X	V1-V3	0%	
SRS016095	764588959	stool	X	V1-V3	0%	
SRS016203	764649650	stool	X	V1-V3	0%	
SRS016267	764669880	stool	X	V3-V5	0%	
SRS019787	764669880	stool	X	V3-V5	0%	
SRS018559	764710340	stool		V3-V5	0%	
SRS062464	764750800	stool		V1-V3	0%	
SRS042703	764811490	stool		V1-V3	0%	
SRS063961	764831721	stool		V3-V5	0%	
SRS063827	764872181	stool		V3-V5	0%	
SRS019910	764892411	stool	X	V3-V5	0%	
SRS063214	764953101	stool		V1-V3	0%	
SRS018733	765013792	stool		V1-V3	0%	
SRS019267	765034022	stool	X	V3-V5	0%	
SRS016335	765074482	stool	X	V1-V3	0%	
SRS049164	765074482	stool	X	V1-V3	0%	
SRS042669	765094712	stool		V1-V3	0%	
SRS018607	765094712	stool		V3-V5	0%	
SRS018817	765135172	stool	X	V3-V5	0%	
SRS018655	765155402	stool		V1-V3	0%	
SRS018712	765195863	stool		V1-V3	0%	
SRS018920	765216093	stool		V1-V3	0%	
SRS018872	765256553	stool		V1-V3	0%	
SRS018968	765276783	stool		V1-V3	0%	
SRS019534	765317243	stool		V1-V3	0%	
SRS019381	765337473	stool		V1-V3	0%	
SRS019846	765661155	stool		V3-V5	0%	
SRS049959	765701615	stool	X	V3-V5	0%	
SRS065421	809635352	stool		V1-V3	0%	
SRS022546	809635352	stool		V3-V5	0%	
SRS048853	840279516	stool		V3-V5	0%	
SRS045607	857980665	stool		V3-V5	0%	
SRS052471	861967750	stool		V3-V5	0%	
SRS042387	863126187	stool		V3-V5	0%	
SRS063068	866653606	stool		V3-V5	0%	
SRS063524	872848689	stool		V3-V5	0%	
SRS023671	892969023	stool		V3-V5	0%	
SRS065500	892969023	stool		V3-V5	0%	
SRS064321	901775393	stool		V1-V3	0%	

Supplementary table 5. Human Microbiome Project (HMP) samples analysed - page 7

SRA accession	Subject	Body Site	WGS	Amplified region	Perc. Brachyspira	Brachyspira in assembly
SRS044146	937415040	stool		V3-V5	0%	
SRS063307	937495960	stool		V3-V5	0%	
SRS049823	953045535	stool		V3-V5	0%	
SRS023242	964271349	stool		V3-V5	0%	
SRS022225	970836795	stool		V3-V5	0%	
SRS050374	970836795	stool		V3-V5	0%	
SRS011061		stool	X			
SRS011084		stool	X			
SRS011134		stool	X			
SRS011239		stool	X			
SRS011302		stool	X			
SRS012273		stool	X			
SRS012902		stool	X			
SRS013158		stool	X			
SRS013215		stool	X			
SRS013476		stool	X			
SRS013521		stool	X			
SRS013800		stool	X			
SRS013951		stool	X			
SRS014313		stool	X			
SRS014459		stool	X			
SRS014683		stool	X			
SRS014979		stool	X			
SRS015065		stool	X			
SRS015217		stool	X			
SRS015264		stool	X			
SRS015369		stool	X			
SRS015794		stool	X			
SRS016989		stool	X			
SRS017247		stool	X			
SRS017433		stool	X			
SRS018351		stool	X			
SRS018575		stool	X			
SRS018656		stool	X			
SRS019030		stool	X			
SRS019161		stool	X			
SRS019397		stool	X			
SRS019601		stool	X			
SRS019968		stool	X			
SRS020328		stool	X			
SRS021948		stool	X			
SRS022071		stool	X			
SRS022137		stool	X			
SRS022524		stool	X			
SRS022713		stool	X			
SRS023346		stool	X			
SRS023526		stool	X			
SRS023583		stool	X			
SRS023829		stool	X			
SRS024009		stool	X			
SRS024075		stool	X			
SRS024132		stool	X			
SRS024435		stool	X			

Supplementary table 5. Human Microbiome Project (HMP) samples analysed - page 8

SRA accession	Subject	Body Site	WGS	Amplified region	Perc. Brachyspira	Brachyspira in assembly
SRS024549		stool	X			
SRS042284		stool	X			
SRS043001		stool	X			
SRS045004		stool	X			
SRS045645		stool	X			
SRS045713		stool	X			
SRS047014		stool	X			
SRS047044		stool	X			
SRS048164		stool	X			
SRS049712		stool	X			
SRS049900		stool	X			
SRS049995		stool	X			
SRS050299		stool	X			
SRS050752		stool	X			
SRS050925		stool	X			
SRS051882		stool	X			
SRS052027		stool	X			
SRS053214		stool	X			
SRS053335		stool	X			
SRS053398		stool	X			
SRS054956		stool	X			
SRS055982		stool	X			
SRS056519		stool	X			
SRS057478		stool	X			
SRS057717		stool	X			
SRS058723		stool	X			
SRS063040		stool	X			
SRS064645		stool	X			
SRS065504		stool	X			
SRS075398		stool	X			
SRS077730		stool	X			
SRS078176		stool	X			

Supplementary table 6. Average Nucleotide Identity between strains of the *Brachyspira* genus

	B. hyodysenteriae NX	PC5099IV	PC3997IV	PC5538III-lc	PC4226IV	*B. hampsonii* 30446	*B. hampsonii* 30599	PC3939II	*B. alvinipulli* ATCC51933-T324	*513A*^{T}
B. hyodysenteriae NX	1.00	0.82	0.83	0.82	0.82	0.89	0.89	0.82	0.87	0.82
PC5099IV	0.82	1.00	0.97	0.97	1.00	0.82	0.82	0.97	0.82	0.97
PC3997IV	0.83	0.97	1.00	0.97	0.97	0.82	0.82	0.97	0.82	0.97
PC5538III-lc	0.82	0.97	0.97	1.00	0.97	0.82	0.82	0.97	0.82	0.97
PC4226IV	0.82	1.00	0.97	0.97	1.00	0.82	0.82	0.97	0.82	0.97
B. hampsonii 30446	0.89	0.82	0.82	0.82	0.82	1.00	0.94	0.82	0.86	0.82
B. hampsonii 30599	0.89	0.82	0.82	0.82	0.82	0.94	1.00	0.82	0.86	0.82
PC3939II	0.82	0.97	0.97	0.97	0.97	0.82	0.82	1.00	0.82	0.97
B. alvinipulli ATCC51933-T324	0.87	0.82	0.82	0.82	0.82	0.86	0.86	0.82	1.00	0.82
513A^T	0.82	0.97	0.97	0.97	0.97	0.82	0.82	0.97	0.82	1.00
B. suanatina AN4859-03	0.93	0.82	0.82	0.82	0.82	0.89	0.89	0.82	0.87	0.82
B. sp G79	0.86	0.83	0.83	0.82	0.83	0.87	0.88	0.83	0.86	0.83
PC5538III-hc	0.86	0.83	0.83	0.82	0.83	0.86	0.86	0.83	0.85	0.82
PC2777IV	0.82	0.97	0.97	0.97	0.97	0.82	0.82	0.97	0.82	0.97
B. pilosicoli P43/6/78	0.86	0.83	0.83	0.83	0.83	0.85	0.86	0.83	0.86	0.83
B. hampsonii NSH-24	0.89	0.82	0.82	0.82	0.82	1.00	0.94	0.82	0.86	0.82
PC5587-u	0.82	0.99	0.97	0.97	0.99	0.82	0.82	0.97	0.82	0.97
PC3714II	0.82	0.97	0.97	0.97	0.97	0.82	0.82	0.97	0.82	0.97
B. hyodysenteriae 865	0.99	0.82	0.82	0.82	0.82	0.89	0.89	0.82	0.87	0.82
B. pilosicoli B2904	0.86	0.83	0.83	0.82	0.83	0.85	0.86	0.83	0.86	0.82
B. sp CAG-700	0.82	0.99	0.97	0.97	0.99	0.82	0.82	0.97	0.82	0.97
B. hampsonii NSH-16	0.89	0.82	0.82	0.82	0.82	0.94	1.00	0.82	0.86	0.82
PC3517II	0.82	0.97	0.99	0.97	0.97	0.82	0.82	0.97	0.82	0.97
PC390II	0.82	0.99	0.97	0.97	0.99	0.82	0.82	0.97	0.82	0.97
PC4597II	0.82	0.99	0.97	0.97	0.99	0.82	0.82	0.97	0.82	0.97
B. pilosicoli 95-1000	0.86	0.83	0.83	0.83	0.83	0.85	0.86	0.83	0.86	0.82
B. hampsonii P280-1	0.89	0.82	0.82	0.82	0.82	0.95	0.94	0.82	0.86	0.82
B. hyodysenteriae WA1	0.99	0.82	0.82	0.82	0.82	0.89	0.89	0.82	0.87	0.82
PC4580III	0.82	0.97	0.97	0.97	0.97	0.82	0.82	0.97	0.82	0.97
B. intermedia PWS-A	0.92	0.82	0.82	0.82	0.82	0.89	0.89	0.82	0.87	0.82
B. pilosicoli WesB	0.86	0.83	0.83	0.82	0.83	0.85	0.86	0.83	0.86	0.82
B. hyodysenteriae FMV89-3323	0.99	0.82	0.82	0.82	0.82	0.89	0.89	0.82	0.87	0.82
B. innocens ATCC29796, B256-F811	0.86	0.82	0.82	0.82	0.82	0.88	0.88	0.82	0.86	0.82
PC5587-p	0.82	0.99	0.97	0.97	0.99	0.82	0.82	0.97	0.82	0.97
PC2022III	0.82	0.97	0.97	0.97	0.97	0.82	0.82	0.97	0.82	0.97
W1	0.82	0.97	0.97	0.97	0.97	0.82	0.82	0.97	0.82	0.97
B. murdochii DSM12563	0.86	0.82	0.82	0.82	0.82	0.88	0.88	0.82	0.86	0.82
PC3053II	0.82	0.97	0.97	0.97	0.97	0.82	0.82	0.97	0.82	0.97
B. hyodysenteriae ATCC27164	0.99	0.82	0.82	0.82	0.82	0.89	0.89	0.82	0.87	0.82

Supplementary table 6. Average Nucleotide Identity between strains of the *Brachyspira* genus- page 2

	B. suanatina AN4859-03	B. sp G79	PC5538III-hc	PC2777IV	*B. pilosicoli* P43/6/78	*B. hampsonii* NSH-24	PC5587-u	PC3714II	*B. hyodysenteriae* 865	*B. pilosicoli* B2904
B. hyodysenteriae NX	0.93	0.86	0.86	0.82	0.86	0.89	0.82	0.82	0.99	0.86
PC5099IV	0.82	0.83	0.83	0.97	0.83	0.82	0.99	0.97	0.82	0.83
PC3997IV	0.82	0.83	0.83	0.97	0.83	0.82	0.97	0.97	0.82	0.83
PC5538III-lc	0.82	0.82	0.82	0.97	0.83	0.82	0.97	0.97	0.82	0.82
PC4226IV	0.82	0.83	0.83	0.97	0.83	0.82	0.99	0.97	0.82	0.83
B. hampsonii 30446	0.89	0.87	0.86	0.82	0.85	1.00	0.82	0.82	0.89	0.85
B. hampsonii 30599	0.89	0.88	0.86	0.82	0.86	0.94	0.82	0.82	0.89	0.86
PC3939II	0.82	0.83	0.83	0.97	0.83	0.82	0.97	0.97	0.82	0.83
B. alvinipulli ATCC51933-T324	0.87	0.86	0.85	0.82	0.86	0.86	0.82	0.82	0.87	0.86
513A^T	0.82	0.83	0.82	0.97	0.83	0.82	0.97	0.97	0.82	0.82
B. suanatina AN4859-03	1.00	0.86	0.86	0.82	0.86	0.89	0.82	0.82	0.93	0.86
B. sp G79	0.86	1.00	0.85	0.83	0.85	0.87	0.83	0.83	0.86	0.86
PC5538III-hc	0.86	0.85	1.00	0.83	0.92	0.86	0.82	0.83	0.86	0.92
PC2777IV	0.82	0.83	0.83	1.00	0.83	0.83	0.97	0.97	0.82	0.83
B. pilosicoli P43/6/78	0.86	0.85	0.92	0.83	1.00	0.85	0.83	0.83	0.86	0.98
B. hampsonii NSH-24	0.89	0.87	0.86	0.83	0.85	1.00	0.82	0.83	0.89	0.86
PC5587-u	0.82	0.83	0.82	0.97	0.83	0.82	1.00	0.97	0.82	0.82
PC3714II	0.82	0.83	0.83	0.97	0.83	0.83	0.97	1.00	0.82	0.82
B. hyodysenteriae 865	0.93	0.86	0.86	0.82	0.86	0.89	0.82	0.82	1.00	0.86
B. pilosicoli B2904	0.86	0.86	0.92	0.83	0.98	0.86	0.82	0.82	0.86	1.00
B. sp CAG-700	0.82	0.83	0.83	0.97	0.83	0.82	0.99	0.97	0.82	0.83
B. hampsonii NSH-16	0.89	0.87	0.86	0.83	0.86	0.94	0.82	0.82	0.89	0.86
PC3517II	0.82	0.83	0.83	0.97	0.83	0.82	0.97	0.97	0.82	0.83
PC390II	0.82	0.83	0.82	0.97	0.83	0.82	1.00	0.97	0.82	0.82
PC4597II	0.82	0.83	0.83	0.97	0.83	0.82	0.99	0.97	0.82	0.83
B. pilosicoli 95-1000	0.86	0.85	0.92	0.83	0.99	0.86	0.82	0.83	0.86	0.98
B. hampsonii P280-1	0.89	0.87	0.86	0.83	0.85	0.95	0.82	0.82	0.89	0.86
B. hyodysenteriae WA1	0.93	0.86	0.86	0.82	0.86	0.89	0.82	0.82	0.99	0.86
PC4580III	0.82	0.83	0.83	0.97	0.83	0.82	0.97	0.97	0.82	0.83
B. intermedia PWS-A	0.93	0.86	0.86	0.82	0.87	0.89	0.82	0.82	0.92	0.87
B. pilosicoli WesB	0.86	0.85	0.92	0.83	0.98	0.86	0.82	0.82	0.86	0.98
B. hyodysenteriae FMV89-3323	0.93	0.86	0.86	0.82	0.86	0.89	0.82	0.82	0.99	0.86
B. innocens ATCC29796, B256-F811	0.86	0.93	0.86	0.82	0.86	0.88	0.83	0.82	0.86	0.86
PC5587-p	0.82	0.83	0.82	0.97	0.83	0.82	1.00	0.97	0.82	0.82
PC2022III	0.82	0.83	0.83	0.97	0.83	0.83	0.97	0.97	0.82	0.82
W1	0.82	0.83	0.83	0.97	0.83	0.82	0.97	0.97	0.82	0.82
B. murdochii DSM12563	0.86	0.93	0.85	0.82	0.85	0.88	0.82	0.82	0.86	0.85
PC3053II	0.82	0.83	0.83	0.97	0.83	0.82	0.97	0.97	0.82	0.83
B. hyodysenteriae ATCC27164	0.93	0.86	0.86	0.82	0.86	0.89	0.82	0.82	0.99	0.86

Supplementary table 6. Average Nucleotide Identity between strains of the *Brachyspira* genus- page 3

	B. sp. CAG-700	*B. hampsonii* NSH-16	PC3517II	PC390II	PC4597II	*B. pilosicoli* 95-1000	*B. hampsonii* P280-1	*B. hyodysenteriae* WA1	PC4580III	*B. intermedia* PWS-A
B. hyodysenteriae NX	0.82	0.89	0.82	0.82	0.82	0.86	0.89	0.99	0.82	0.92
PC5099IV	0.99	0.82	0.97	0.99	0.99	0.83	0.82	0.82	0.97	0.82
PC3997IV	0.97	0.82	0.99	0.97	0.97	0.83	0.82	0.82	0.97	0.82
PC5538III-lc	0.97	0.82	0.97	0.97	0.97	0.83	0.82	0.82	0.97	0.82
PC4226IV	0.99	0.82	0.97	0.99	0.99	0.83	0.82	0.82	0.97	0.82
B. hampsonii 30446	0.82	0.94	0.82	0.82	0.82	0.85	0.95	0.89	0.82	0.89
B. hampsonii 30599	0.82	1.00	0.82	0.82	0.82	0.86	0.94	0.89	0.82	0.89
PC3939II	0.97	0.82	0.97	0.97	0.97	0.83	0.82	0.82	0.97	0.82
B. alvinipulli ATCC51933-T324	0.82	0.86	0.82	0.82	0.82	0.86	0.86	0.87	0.82	0.87
513A^{T}	0.97	0.82	0.97	0.97	0.97	0.82	0.82	0.82	0.97	0.82
B. suanatina AN4859-03	0.82	0.89	0.82	0.82	0.82	0.86	0.89	0.93	0.82	0.93
B. sp G79	0.83	0.87	0.83	0.83	0.83	0.85	0.87	0.86	0.83	0.86
PC5538III-hc	0.83	0.86	0.83	0.82	0.83	0.92	0.86	0.86	0.83	0.86
PC2777IV	0.97	0.83	0.97	0.97	0.97	0.83	0.83	0.82	0.97	0.82
B. pilosicoli P43/6/78	0.83	0.86	0.83	0.83	0.83	0.99	0.85	0.86	0.83	0.87
B. hampsonii NSH-24	0.82	0.94	0.82	0.82	0.82	0.86	0.95	0.89	0.82	0.89
PC5587-u	0.99	0.82	0.97	1.00	0.99	0.82	0.82	0.82	0.97	0.82
PC3714II	0.97	0.82	0.97	0.97	0.97	0.83	0.82	0.82	0.97	0.82
B. hyodysenteriae 865	0.82	0.89	0.82	0.82	0.82	0.86	0.89	0.99	0.82	0.92
B. pilosicoli B2904	0.83	0.86	0.83	0.82	0.83	0.98	0.86	0.86	0.83	0.87
B. sp CAG-700	1.00	0.82	0.97	0.99	0.99	0.83	0.82	0.82	0.97	0.82
B. hampsonii NSH-16	0.82	1.00	0.82	0.82	0.82	0.86	0.94	0.89	0.82	0.89
PC3517II	0.97	0.82	1.00	0.97	0.97	0.83	0.82	0.82	0.97	0.82
PC390II	0.99	0.82	0.97	1.00	0.99	0.82	0.82	0.82	0.97	0.82
PC4597II	0.99	0.82	0.97	0.99	1.00	0.82	0.82	0.82	0.97	0.82
B. pilosicoli 95-1000	0.83	0.86	0.83	0.82	0.82	1.00	0.86	0.86	0.83	0.87
B. hampsonii P280-1	0.82	0.94	0.82	0.82	0.82	0.86	1.00	0.89	0.82	0.89
B. hyodysenteriae WA1	0.82	0.89	0.82	0.82	0.82	0.86	0.89	1.00	0.82	0.92
PC4580III	0.97	0.82	0.97	0.97	0.97	0.83	0.82	0.82	1.00	0.82
B. intermedia PWS-A	0.82	0.89	0.82	0.82	0.82	0.87	0.89	0.92	0.82	1.00
B. pilosicoli WesB	0.83	0.86	0.83	0.82	0.83	0.98	0.86	0.86	0.83	0.86
B. hyodysenteriae FMV89-3323	0.82	0.89	0.82	0.82	0.82	0.86	0.89	0.99	0.82	0.92
B. innocens ATCC29796, B256-F811	0.82	0.88	0.82	0.83	0.82	0.86	0.88	0.86	0.82	0.86
PC5587-p	0.99	0.82	0.97	1.00	0.99	0.82	0.82	0.82	0.97	0.82
PC2022III	0.97	0.82	0.97	0.97	0.97	0.83	0.82	0.82	0.98	0.82
W1	0.97	0.82	0.97	0.97	0.97	0.83	0.82	0.82	0.97	0.82
B. murdochii DSM12563	0.82	0.88	0.83	0.83	0.82	0.86	0.88	0.86	0.83	0.86
PC3053II	0.97	0.82	0.97	0.97	0.97	0.83	0.82	0.82	0.97	0.82
B. hyodysenteriae ATCC27164	0.82	0.89	0.82	0.82	0.82	0.86	0.89	0.99	0.82	0.92

Supplementary table 6. Average Nucleotide Identity between strains of the *Brachyspira* genus- page 2

	B. pilosicoli WesB	*B. hyodysenteriae* FMV89-3323	*B. innocens* ATCC29796, B256-F811	PC5587-p	PC2022III	W1	*B. murdochii* DSM12563	PC3053II	*B. hyodysenteriae* ATCC27164
B. hyodysenteriae NX	0.86	0.99	0.86	0.82	0.82	0.82	0.86	0.82	0.99
PC5099IV	0.83	0.82	0.82	0.99	0.97	0.97	0.82	0.97	0.82
PC3997IV	0.83	0.82	0.82	0.97	0.97	0.97	0.82	0.97	0.82
PC5538III-lc	0.82	0.82	0.82	0.97	0.97	0.97	0.82	0.97	0.82
PC4226IV	0.83	0.82	0.82	0.99	0.97	0.97	0.82	0.97	0.82
B. hampsonii 30446	0.85	0.89	0.88	0.82	0.82	0.82	0.88	0.82	0.89
B. hampsonii 30599	0.86	0.89	0.88	0.82	0.82	0.82	0.88	0.82	0.89
PC3939II	0.83	0.82	0.82	0.97	0.97	0.97	0.82	0.97	0.82
B. alvinipulli ATCC51933-T324	0.86	0.87	0.86	0.82	0.82	0.82	0.86	0.82	0.87
513A^{T}	0.82	0.82	0.82	0.97	0.97	0.97	0.82	0.97	0.82
B. suanatina AN4859-03	0.86	0.93	0.86	0.82	0.82	0.82	0.86	0.82	0.93
B. sp G79	0.85	0.86	0.93	0.83	0.83	0.83	0.93	0.83	0.86
PC5538III-hc	0.92	0.86	0.86	0.82	0.83	0.83	0.85	0.83	0.86
PC2777IV	0.83	0.82	0.82	0.97	0.97	0.97	0.82	0.97	0.82
B. pilosicoli P43/6/78	0.98	0.86	0.86	0.83	0.83	0.83	0.85	0.83	0.86
B. hampsonii NSH-24	0.86	0.89	0.88	0.82	0.83	0.82	0.88	0.82	0.89
PC5587-u	0.82	0.82	0.83	1.00	0.97	0.97	0.82	0.97	0.82
PC3714II	0.82	0.82	0.82	0.97	0.97	0.97	0.82	0.97	0.82
B. hyodysenteriae 865	0.86	0.99	0.86	0.82	0.82	0.82	0.86	0.82	0.99
B. pilosicoli B2904	0.98	0.86	0.86	0.82	0.82	0.82	0.85	0.83	0.86
B. sp CAG-700	0.83	0.82	0.82	0.99	0.97	0.97	0.82	0.97	0.82
B. hampsonii NSH-16	0.86	0.89	0.88	0.82	0.82	0.82	0.88	0.82	0.89
PC3517II	0.83	0.82	0.82	0.97	0.97	0.97	0.83	0.97	0.82
PC390II	0.82	0.82	0.83	1.00	0.97	0.97	0.83	0.97	0.82
PC4597II	0.83	0.82	0.82	0.99	0.97	0.97	0.82	0.97	0.82
B. pilosicoli 95-1000	0.98	0.86	0.86	0.82	0.83	0.83	0.86	0.83	0.86
B. hampsonii P280-1	0.86	0.89	0.88	0.82	0.82	0.82	0.88	0.82	0.89
B. hyodysenteriae WA1	0.86	0.99	0.86	0.82	0.82	0.82	0.86	0.82	0.99
PC4580III	0.83	0.82	0.82	0.97	0.98	0.97	0.83	0.97	0.82
B. intermedia PWS-A	0.86	0.92	0.86	0.82	0.82	0.82	0.86	0.82	0.92
B. pilosicoli WesB	1.00	0.86	0.86	0.82	0.83	0.82	0.86	0.82	0.86
B. hyodysenteriae FMV89-3323	0.86	1.00	0.86	0.82	0.82	0.82	0.86	0.82	0.99
B. innocens ATCC29796, B256-F811	0.86	0.86	1.00	0.83	0.82	0.82	0.93	0.82	0.86
PC5587-p	0.82	0.82	0.83	1.00	0.97	0.97	0.82	0.97	0.82
PC2022III	0.83	0.82	0.82	0.97	1.00	0.97	0.82	0.97	0.82
W1	0.82	0.82	0.82	0.97	0.97	1.00	0.82	0.97	0.82
B. murdochii DSM12563	0.86	0.86	0.93	0.82	0.82	0.82	1.00	0.83	0.86
PC3053II	0.82	0.82	0.82	0.97	0.97	0.97	0.83	1.00	0.82
B. hyodysenteriae ATCC27164	0.86	0.99	0.86	0.82	0.82	0.82	0.86	0.82	1.00

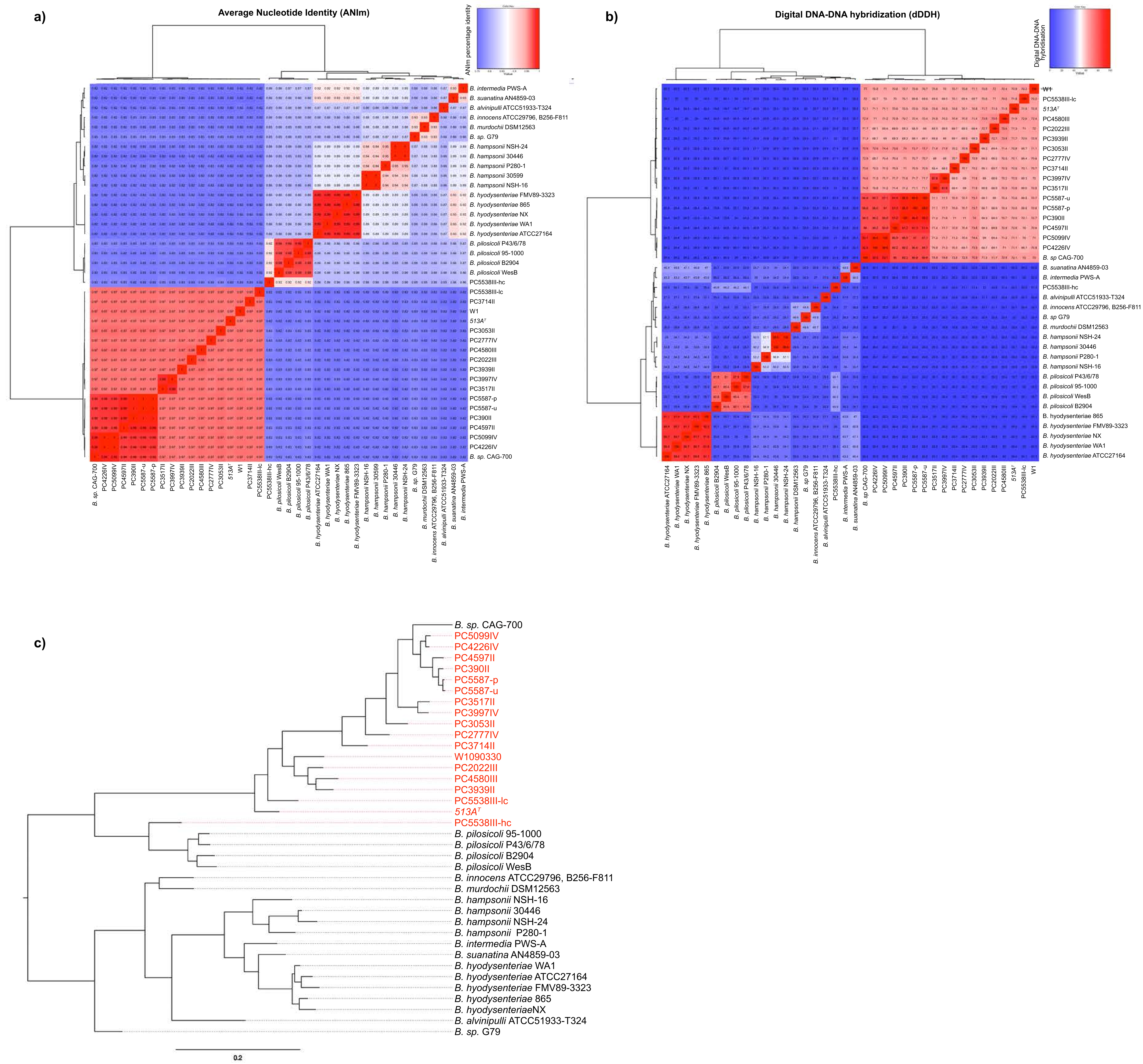

Figure S1. Comparative genomics within the *Brachyspira* genus. A) Average Nucleotide Identity (ANI) comparison. B) Digital DNA-DNA Hybridisation (dDDH) comparison. C) Tree based on accessory genome analysis (presence/absence of accessory genes).

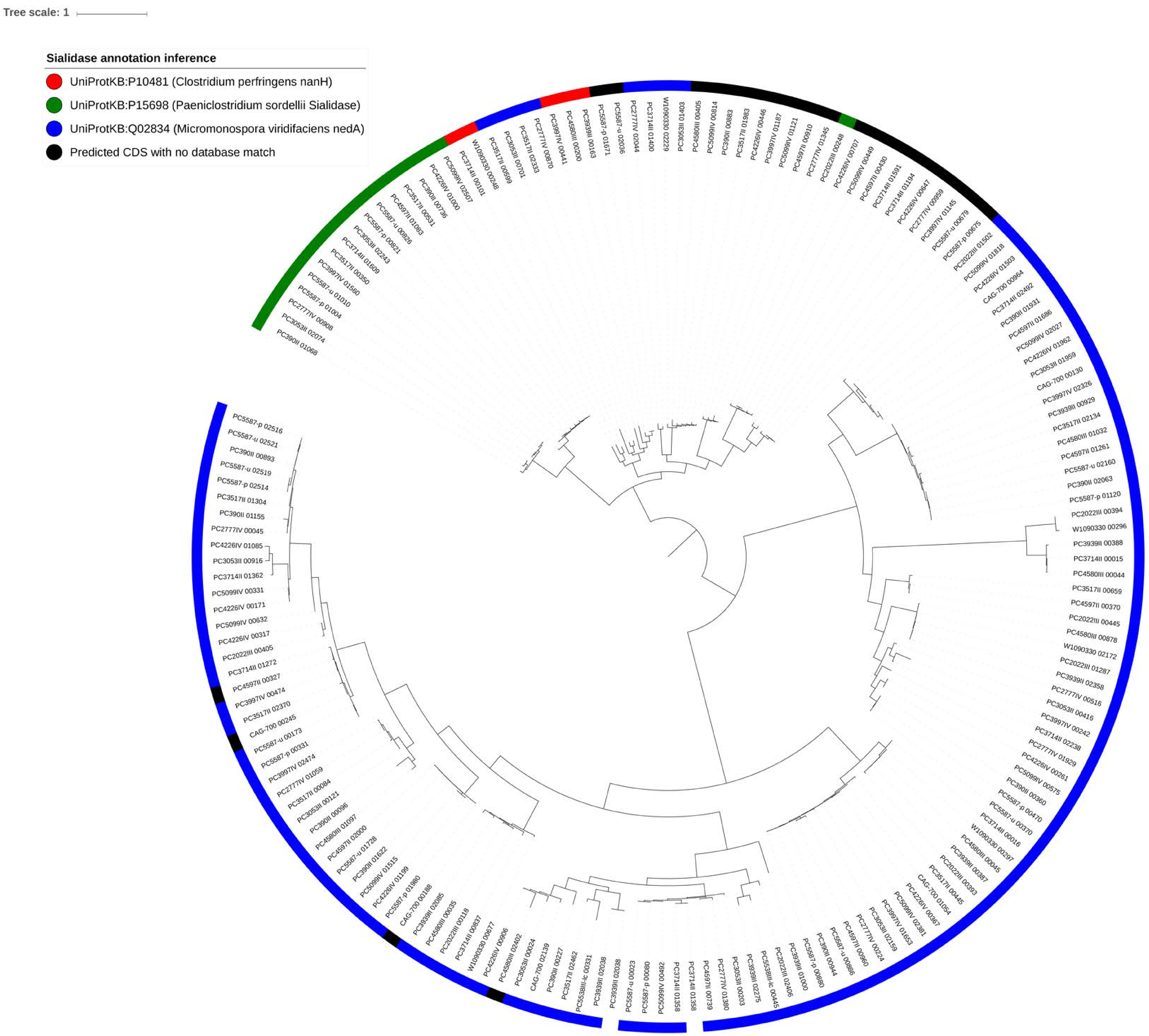

Strain	# sialidase genes
PC3714II	13
PC4226IV	13
PC3517II	12
PC390II	12
PC5099IV	12
PC5587-p	12
PC5587-u	12
PC2777IV	11
PC3053II	11
PC4597II	10
PC2022III	9
PC3939II	9
PC3997IV	9
PC4580III	9
CAG-700	6
W1	6
PC5538III-lc	2
513A	0

Figure S2. Maximum Likelihood tree of the genes annotated to encode for Sialidase in the *B. aalborgi* genomes. Leaves are coloured according to the source of annotation inference and the table is summarizing the number of sialidase genes per isolate.

a)

b)

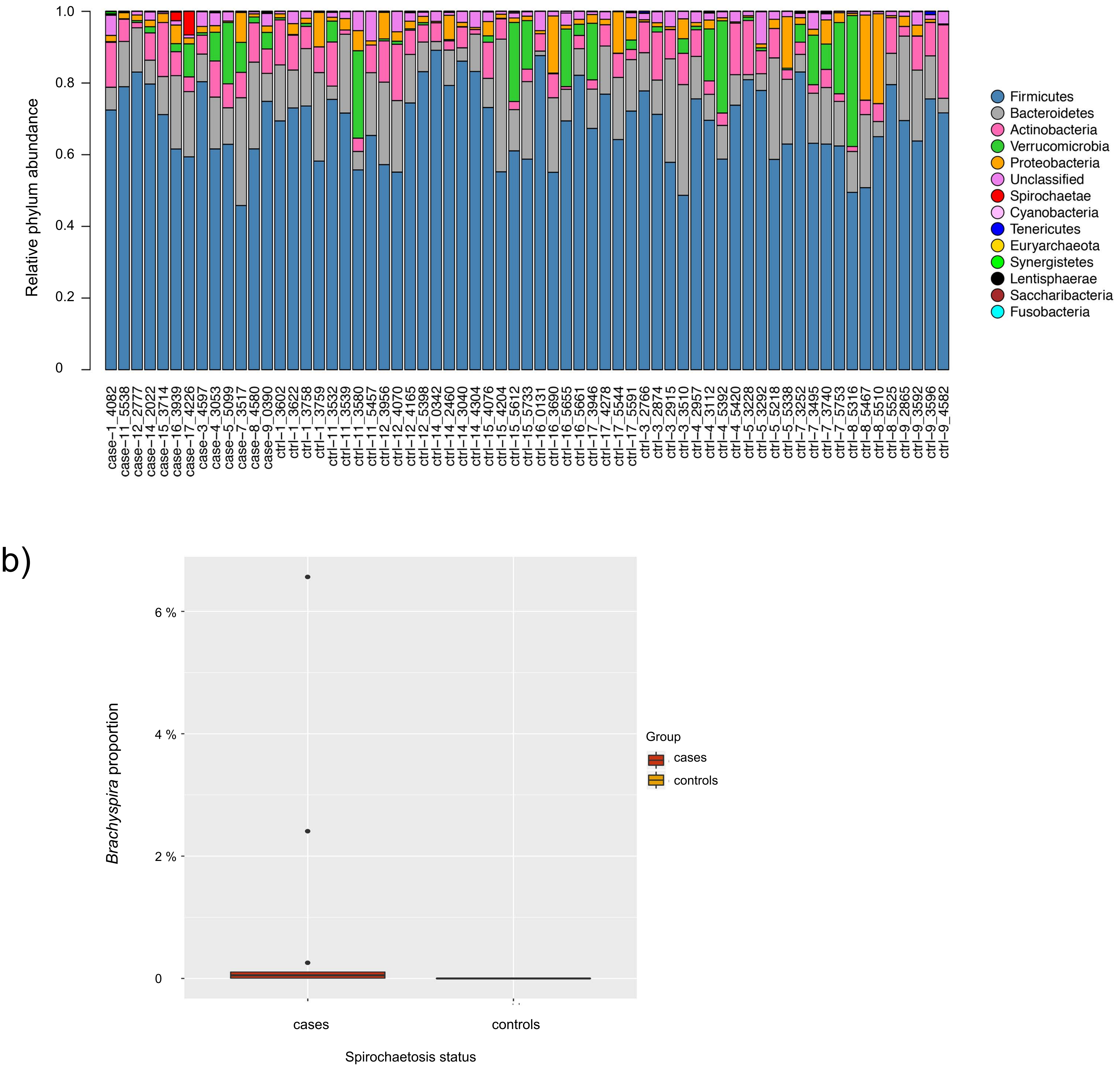

Figure S3. 16S amplicon-based microbiota analysis in spirochaetosis cases and unaffected controls. A) Relative phylum composition in cases and controls. B) Proportion of reads assigned to the *Brachyspira* genus, cases and controls.

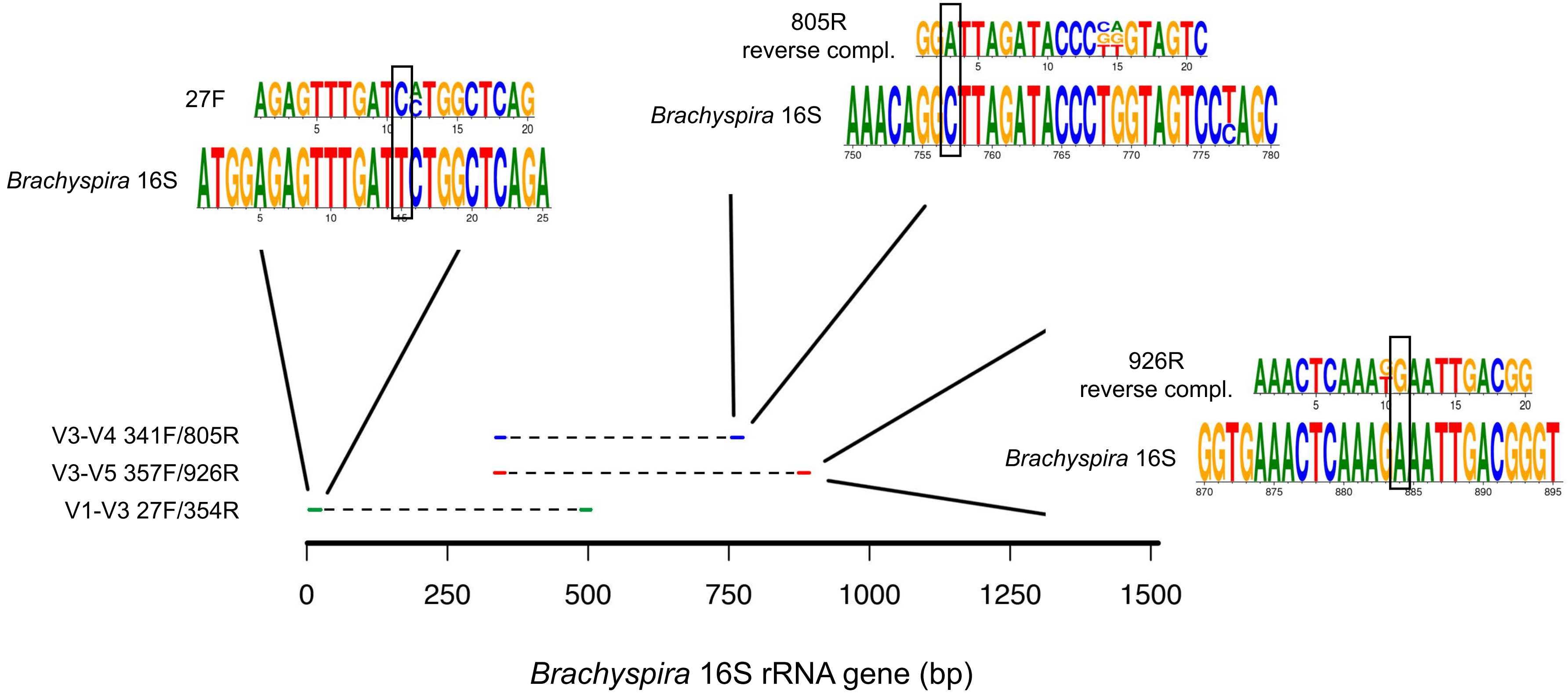

Figure S4. Primer mismatches in the different primer combinations compared to *Brachyspira* 16S rRNA sequences. Magnified areas show WebLogo representation of the *Brachyspira* 16S alignment in the primer target regions, together with the primers in question. Boxes mark the bases with mismatches.

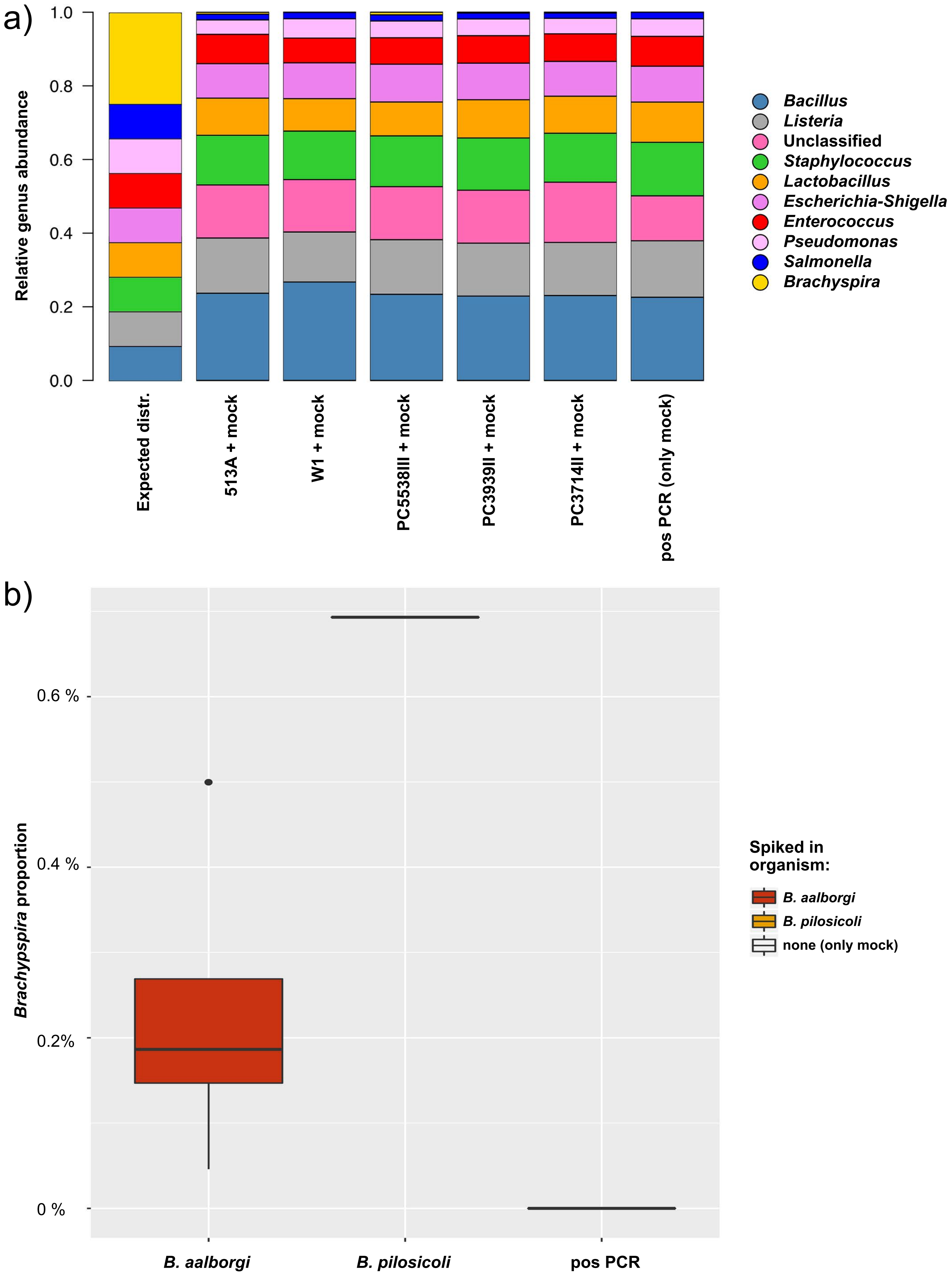

Figure S5. Verification of primer failure in samples of known compositions. All samples were containing a 1:3 proportion of pure *Brachyspira* DNA and a mock community. A) Phylum composition in the different samples. B) Proportion of reads classified to the *Brachyspira* genus depending on the *Brachyspira* species spiked in. The left-most column represents the expected distribution.

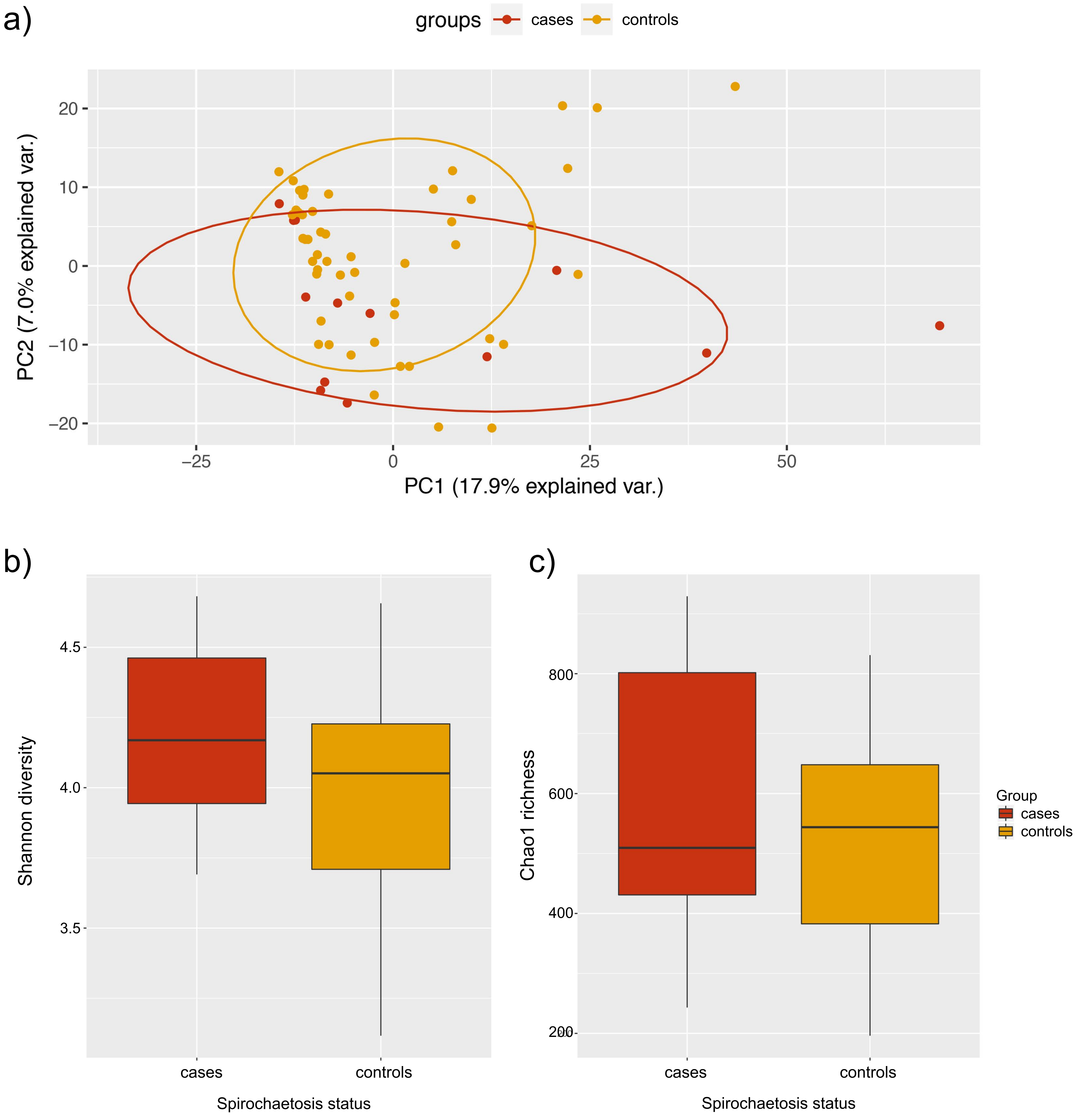

Figure S6. 16S amplicon-based microbiota analysis in spirochaetosis cases and unaffected controls. A) Principle component analysis of OTU composition in cases and controls. B) Shannon & Chao1 diversity

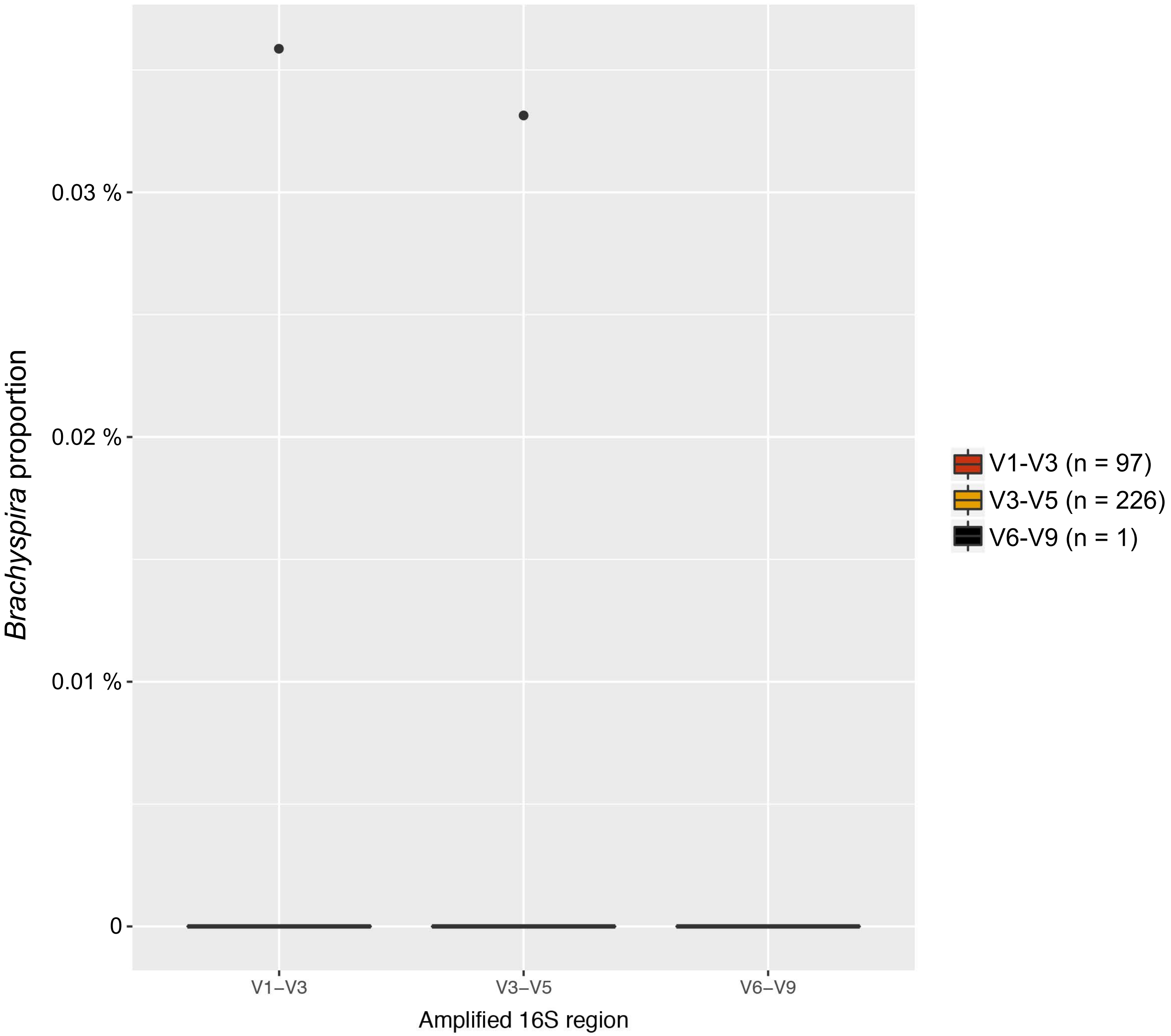

Figure S7. Proportion of reads assigned to the *Brachyspira* genus in 324 individuals from the Human Microbiome Project (HMP).
